# Absence of long-term balancing selection on variation in *EuMYB3*, an *R2R3-MYB* gene responsible for the anther-color polymorphism in *Erythronium umbilicatum*

**DOI:** 10.1038/s41598-024-56117-y

**Published:** 2024-03-04

**Authors:** Rong-Chien Lin, Mark D. Rausher

**Affiliations:** 1 Department of Biology, Duke University, Durham, NC 27708 USA; 2 Department of Ecology and Evolutionary Biology, University of Connecticut, Storrs, CT 06269 USA

**Keywords:** Polymorphism, Anther color, Anthocyanin, *Erythronium*, R2R3-MYB, Balancing selection, Plant evolution, Evolutionary biology

## Abstract

Balancing selection has been shown to be common in plants for several different types of traits, such as self-incompatibility and heterostyly. Generally, for these traits balancing selection is generated by interactions among individuals or between individuals and other species (e.g., pathogens or pollinators). However, there are phenotypic polymorphisms in plants that do not obviously involve types of interactions that generate balancing selection. Little is known about the extent to which balancing selection also acts to preserve these polymorphisms. Here we ask whether balancing selection preserves an anther-color polymorphism in *Erythronium umbilicatum* (Liliaceae)*.* We identified a major gene underlying this polymorphism. We then attempted to detect signatures of balancing selection on that gene by developing a new coalescence test for balancing selection. We found that variation in anther color is in large part caused by variation in a paralog of *EuMYB3*, an anthocyanin-regulating *R2R3-MYB* transcription factor. However, we found little evidence for balancing selection having acted historically on *EuMYB3*. Our results thus suggest that plant polymorphisms, especially those not involved in interactions that are likely to generate negative frequency-dependent selection, may reflect a transient state in which one morph will eventually be fixed by either genetic drift or directional selection. Our results also suggest that regulation of the anthocyanin pathway is more evolutionarily labile than is generally believed.

## Introduction

Accounting for the presence and persistence of genetic polymorphisms, and genetic variation more generally, has been a central theme in evolutionary biology for almost three-quarters of a century (see Ref.^1^ for a historical account). Polymorphisms may reflect transient conditions, such as when alternative alleles are selectively neutral or when an advantageous allele is replacing a disadvantageous allele. By contrast, various types of balancing selection (e.g., heterozygote advantage, negative frequency-dependent selection, spatial and/or temporal variation in selection) can in theory maintain polymorphisms for extended periods, or even indefinitely.

In plants, there is substantial evidence for the operation of balancing selection^1^. Examples include maintenance of self-incompatibility alleles^2^, nuclear-cytoplasmic gynodioecy^3,4^, heterostyly^5^, flower-color polymorphisms^6,7^, and polymorphisms at loci affecting host–pathogen interactions^8^. Most of these examples involve negative frequency-dependent selection generated by interactions among individuals in the population (e.g., self-incompatibility, nuclear-cytoplasmic gynodioecy), by interactions between plants and their pollinators (heterostyly, flower-color polymorphisms), or by interactions between plants and their herbivores and pathogens^9^.

In an extensive survey, Gottlieb^10^ provided evidence that plant populations harbor many polymorphisms for morphological characters that are controlled by one or two loci. Most of these do not obviously fall into any of the categories described above, and also are not obviously involved with interactions generating negative frequency-dependent selection, or interactions subject to spatial/temporal variation in selection. Whether balancing selection contributes to these types of characters is thus a largely unanswered question.

**Figure 1:**
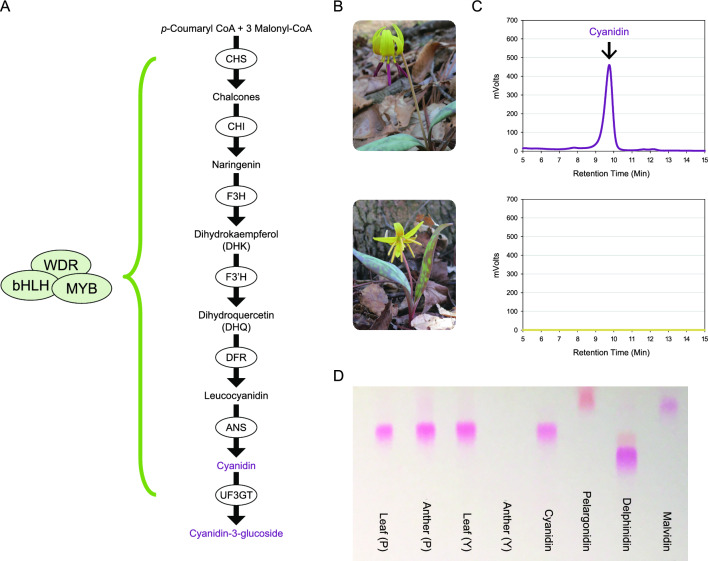
(**A**) A schematic diagram of the anthocyanin biosynthetic pathway. Enzymes are shown in circles: *CHS* chalcone synthase, *CHI* chalcone isomerase, *F3H* flavanone-3-hydroxylase, *F3ʹH* flavonoid 3ʹ-hydroxylase, *F3ʹ5ʹH* flavonoid 3ʹ,5ʹ-hydroxylase, *DFR* dihydroflavonol-4-reductase, *ANS* anthocyanidin synthase, *UF3GT* UDP-flavonoid-3-*O*-glucosyl-transferase. The pathway is regulated by a protein complex composed of the MYB, bHLH and WDR transcription factors. (**B**) Purple-anthered and yellow-anthered *Erythronium umbilicatum*. (**C**) HPLC traces of anthocyanidins extracted from the purple and yellow anthers of *E*. *umbilicatum*. (**D**) Forestal chromatogram of anthocyanidins extracted from the anthers and leaves of the same *E*. *umbilicatum* plants (P: purple-anthered; Y: yellow-anthered). Extracts and standards (cyanidin, pelargonidin, delphinidin, and malvidin) were run together on a TLC plate.

One such character is a well-known anther-color polymorphism in several species in the genus *Erythronium* (Liliaceae). In the species *E. grandiflorum*, *E. americanum*, and *E. umbilicatum*, one morph has purple or red anthers and pollen that contain anthocyanins, while the other morph is yellow and anthocyanin-less (see below). The possibility that this polymorphism may be trans-specific suggests that there may have been long-term balancing selection acting to maintain it. One study on *E. americanum* found that some pollinators form preferences for one morph over the other^11^. However, it is not known whether these preferences generate negative frequency-dependent selection. For other species, possible factors contributing to maintenance of the polymorphism have not been examined. In this study, we investigate the polymorphism in *E. umbilicatum*. *E. umbilicatum* is a long-lived, spring ephemeral that reproduces multiple times throughout its lifetime. Reproductive plants produce two leaves and a single yellow, hermaphroditic flower. The green leaves are often irregularly mottled with brown-purple splotches that contain anthocyanins (see “Results” section).* E*. *umbilicatum* occurs commonly in the deciduous forest in the southeastern United States^12^ and is largely self-incompatible^13^. The diploid *E*. *umbilicatum* lacks stolons^12^, which suggests that clonal reproduction is relatively uncommon in this species. The purple-anthered and yellow-anthered *E*. *umbilicatum* (hereafter “purple plants” or “purple individuals” and “yellow plants” or “yellow individuals”) (Fig. 1B) were both observed in all 16 surveyed populations in North Carolina, USA, with the purple morph always being more common (frequency 80–96%; Supplementary Table S1).

Historically, two approaches have been taken to detect balancing selection on polymorphisms. One is quantification of patterns of selection acting on phenotypic variants^6,7^. The other is attempting to detect signatures of balancing selection on the gene responsible for the polymorphism^14^, which requires first to identify the causal gene and such a process is often costly and time-consuming. Here we adopt this second approach. Specifically, we identified a gene associated with the anther-color polymorphism (“causal gene”) in *E. umbilicatum* by examining the biochemical and genetic basis of anther colors. Unfortunately, because *Erythronium* species are non-model organisms with long generation time (typically > 5 years)^15^, we are unable to perform crosses and examine co-segregation of individual genes with anther colors (e.g., Refs.^16,17^). Moreover, functional tests, for example, gene transformation, viral induced gene silencing (VIGS), and CRISPER/CAS9 modification, are not accessible for the *Erythronium* species. Instead, to identify the gene most likely responsible for the anther-color polymorphism, we quantified expression of genes that are involved in anthocyanin pigmentation. Anthocyanin pigments are produced by the anthocyanin biosynthetic pathway (ABP) (Fig. 1), which is controlled by the MYB-bHLH-WDR (MBW) protein complex^18^. This complex may control the entire enzyme-coding genes in the pathway or only a subset of the genes (e.g., Refs.^19,20^). We then showed that downregulation of an *R2R3-MYB* transcription factor (TF) correlates with downregulation of anthocyanin enzyme-coding genes. We also demonstrated that genotype at that TF correlates strongly with anther color, implicating it as a causal gene. Finally, we developed a new coalescent test to detect the operation of balancing selection on this causal gene.

## Results

### Cyanidin-derived anthocyanins occur in the purple anthers only

The purple anthers of *E*. *umbilicatum* have cyanidin-derived anthocyanins. But in the yellow anthers, there are no detectable anthocyanins (Fig. 1C,D). However, despite the absence of anthocyanins in the yellow anthers, cyanidin-derived anthocyanins are present in the leaves of the yellow *E*. *umbilicatum* (Fig. 1D). This observation suggests that the ABP enzyme-coding genes in the yellow plants are functional and expressed in the leaves, and thus changes in their gene expression, rather than changes in functionality, are likely responsible for the anther-color difference.

### *EuDfr*, *EuAns*, *EuUF3GT* and *EubHLH2* are downregulated in the yellow anthers

Our transcriptome data (Table 1) reveal two relevant patterns. First, BLAST searches against the transcriptomes identified twelve ABP genes: single copies of seven enzyme-coding genes (*EuChs*, *EuChi*, *EuF3h*, *EuF3ʹh*, *EuDfr*, *EuAns* and *EuUf3gt*) (see Fig. 1 for full gene names) and five transcription factors (*EuMYB3*, *EubHLH1*, *EubHLH2*, *EuWDR1* and *EuWDR2*). *EuMYB3* belongs to the anthocyanin-regulating subgroup 6 *R2R3-MYB*s^21^ (Supplementary Fig. S1), and the two *bHLH* genes are the members of the subgroup IIIf *bHLH* family, to which most anthocyanin regulators belong^22^ (Supplementary Fig. S2). Second, *EuDfr*, *EuAns*, *EuUf3gt*, *EubHLH1*, and *EubHLH2* are missing in the yellow-anther transcriptome, and analyses of transcript abundance show that *EuDfr*, *EuAns*, *EuUf3gt*, *EubHLH1*, and *EubHLH2* have the FPKM values of zero or very close to zero when mapping the yellow-anther reads to the purple-anther reference. The latter result suggests that expression of *EuDfr*, *EuAns*, *EuUf3gt*, *EubHLH1*, and *EubHLH2* are greatly reduced in the yellow anthers. Although *R3-MYB* repressors have been documented for inhibiting anthocyanin production^23–26^, we were unable to identify any candidates for *R3-MYB*s from our transcriptomes, as the bitscores of the BLAST hits are all less than 150.

**Table 1:** The anthocyanin genes identified from the *Erythronium umbilicatum* transcriptomes.

Contig ID	Gene	BLAST bitscore	Expression (FPKM)	
			Purple anther	Yellow anther
Purple-anther reference				
EuP_33596_c0_g1	*EuChs*	893	11.08	22.98
EuP_37117_c0_g2	*EuChi*	398	17.77	17.46
EuP_36256_c0_g2	*EuF3h*	736	9.15	4.35
EuP_38329_c0_g1	*EuF3'h*	730	11.18	7.55
EuP_39244_c0_g2	*EuDfr*	788	23.53	0.05
EuP_35520_c1_g1	*EuAns*	525	7.76	0.05
EuP_39893_c0_g2	*EuUf3gt*	866	7.85	0.04
EuP_21744_c0_g1	*EubHLH1*	270	1.49	0.32
EuP_52832_c0_g1	*EubHLH2*	219	0.91	0
EuP_30989_c0_g2	*EuWDR1*	394	2.77	1.86
EuP_33697_c0_g1	*EuWDR2*	289	4.45	4.47
Yellow-anther reference				
EuY_40055_c0_g7	*EuChs*	893	13.27	22.02
EuY_38665_c0_g2	*EuChi*	397	21.86	18.34
EuY_25883_c0_g1	*EuF3h*	736	9.49	3.58
EuY_36308_c1_g9	*EuF3'h*	603	6.79	4.08
EuY_17157_c0_g2	*EuMYB3*	280	1.35	1.92
EuY_34064_c1_g2	*EuWDR1*	282	1.91	1.62
EuY_34064_c1_g1	*EuWDR2*	289	5.85	5.01

These genes were identified through BLASTing. Gene expression levels were estimated as FPKM values by mapping reads to the transcriptome references of purple anther and yellow anther, separately.

**Figure 2:**
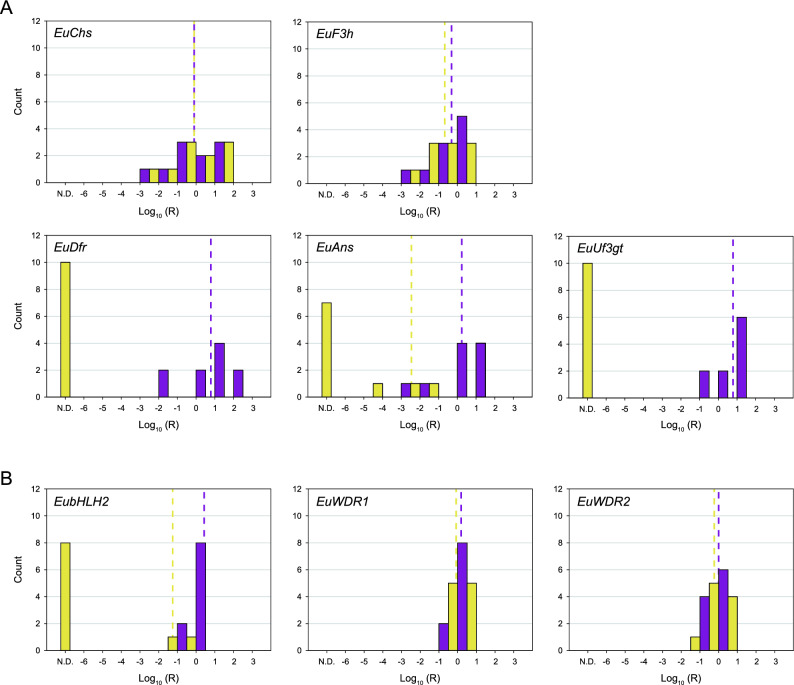
Frequency histograms of relative expression of (**A**) five enzyme-coding genes and (**B**) three transcription factors in the purple and yellow anthers of *E. umblicatum* from qPCR analyses. X-axis indicates the logarithm of the relative expression ratio, Log_10_(R). Relative expression ratios were calculated as described in Supplementary Methods S3. The housekeeping gene *EF1α* (elongation factor 1-alpha) was used as a reference gene for normalizing expression levels across samples. Purple bars represent purple-anthered plants (*N* = 10), and yellow bars represent yellow-anthered plants (*N* = 10). N.D. indicates that the expression was not detectable. Dotted lines indicate the means for each color group.

We further examined this downregulation pattern with qPCR (Fig. 2). The upstream genes *EuChs* and *EuF3h* exhibit no detectable expression differences between the yellow and purple anthers (Student’s *t*-test, *EuChs*: *t* =  − 0.006, *P* = 0.995; *EuF3h*: *t* = 0.913, *P* = 0.373). However, the downstream genes *EuDfr*, *EuAns* and *EuUf3gt* are expressed at greatly reduced levels in the yellow anthers (Mann–Whitney test, *EuDfr*: *U* = 100, *P* < 0.001; *EuAns*: *U* = 97, *P* < 0.001; *EuUf3gt*: *U* = 100, *P* < 0.001).

The qPCR analysis of the identified transcription factors (Fig. 2B) also shows the pattern that is consistent with our transcriptome results. While *EuWDR1* and *EuWDR2* are not expressed differentially between the color morphs (*EuWDR1*: *t* = 1.427, *P* = 0.171; *EuWDR2*: *t* = 1.148, *P* = 0.266), *EubHLH2* is expressed significantly less in the yellow anthers (*EubHLH2*: *U* = 99, *P* < 0.001). We were unable to include *EubHLH1* and *EuMYB3* in the qPCR assays, because amplification of *EubHLH1* has never been successful and multiple copies of *EuMYB3* were detected (see below).

### The downregulated enzyme-coding genes are functional in the yellow-anthered plants

**Figure 3:**
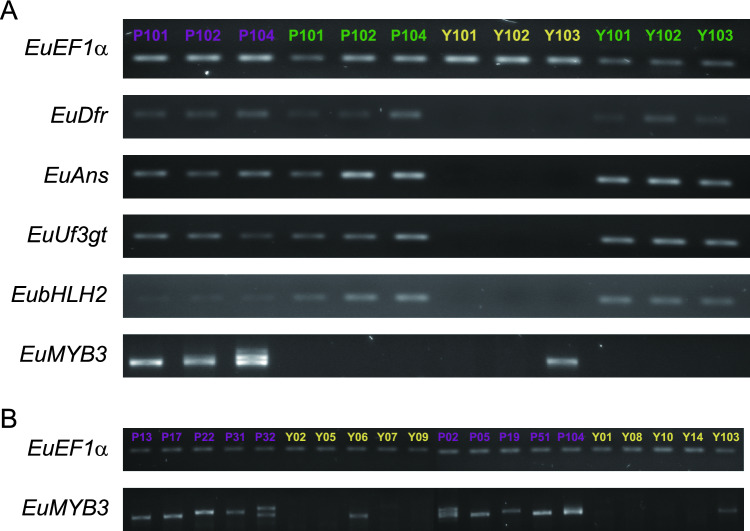
Semi-quantification of gene expression. (**A**) Expression of *EuDfr*, *EuAns*, *EuUf3gt*, *EubHLH2* and *EuMYB3* in the anther and leaf tissues of three purple-anthered and three yellow-anthered *E. umblicatum* plants. For each gene, all the samples were run on the same gel. (**B**) Expression of *EuMYB3* in the anthers of ten purple-anthered and ten yellow-anthered *E. umblicatum* plants. Multiple copies of *EuMYB3* are detected, as shown by multiple bands with different sizes. Plant IDs are shown at the top of each lane: samples of purple anthers are color-coded purple; samples of yellow anthers are color-coded yellow; samples of leaves are color-coded green. A constitutively expressed gene *EuEF1α* was included for cDNA quality control. Two gels were run for each gene: samples P13-Y09 were run on a single gel and samples P02-Y103 were run on the other gel. Full unprocessed gel photographs are shown in Supplementary Figs. S4–S13 and Supplementary Dataset S2.

Although downregulation of *EuDfr*, *EuAns*, and *EuUf3gt* can explain the absence of anthocyanins in yellow anthers, it is also possible that one or more of these enzymes have been functionally inactivated. However, two lines of evidence suggest this possibility is unlikely. First, the sequences of seven enzyme-coding genes (*EuChs*, *EuChi*, *EuF3h*, *EuF3ʹh*, *EuDfr*, *EuAns*, and *EuUf3gt*) from anther RNA are identical to corresponding sequences obtained from leaf RNA of the same plant (GenBank accession numbers: OK648430–OK648447), indicating that the same gene copies are expressed in the anther and leaf tissues. Because we were not able to amplify *EubHLH1* from *E. umbilicatum* and were only able to obtain partial coding sequence of *EubHLH2* from anthers and leaves, we cannot conclusively infer that the same copies of *EubHLH* genes are expressed in the two tissues. Second, given that cyanidin-derived anthocyanins are present in the leaves, these seven enzyme-coding genes should be functional and expressed in the leaves of the yellow plants. Indeed, our semi-quantitative PCR analyses show that *EuDfr*, *EuAns*, *EuUf3gt*, and *EubHLH2* are expressed in the leaf tissue of both purple and yellow plants, although these genes are not expressed in the yellow anthers (Fig. 3A). Consequently, these genes cannot be non-functional in the yellow plants.

### *EuMYB3* represents a family of *R2R3-MYB* genes in *E. umbilicatum*

We identified only a single *R2R3-MYB* gene of subgroup 6 in the anther transcriptomes (i.e., *EuMYB3*; Table 1). EuMYB3 is likely an anthocyanin regulator because it clusters phylogenetically with R2R3-MYB proteins from other species that have been characterized as anthocyanin-regulating proteins (Supplementary Fig. S1). In addition, it has the conserved motif “[K/R]P[R/Q]PR” that is present in all anthocyanin-regulating R2R3-MYBs (motif 6^21^; Supplementary Fig. S3). The expression domain of *EuMYB3* is specific to the anther tissue, with the gene not being expressed in leaves (Fig. 3A). In addition, it is expressed in all examined purple individuals, but largely downregulated in the yellow individuals (Fig. 3B).

When we amplified, cloned and sequenced this gene from gDNA and anther cDNA, however, it became evident that multiple copies of this gene exist in the genome. For example, from gDNA of the yellow individuals Y05 and Y07, we recovered 10 and 9 unique sequences, respectively (Supplementary Table S4). Allowing two sequences per locus, these numbers indicate that there are at least 5 copies of *EuMYB3* in the yellow individuals. We obtained 123 sequences from gDNA, which are distributed over 10 purple and 10 yellow plants, for an average of 6.15 sequences (> 3 paralogs) per individual. Obviously, not all individuals revealed 5 paralogs, especially since the same gene copy may have been amplified multiple times.

**Figure 4:**
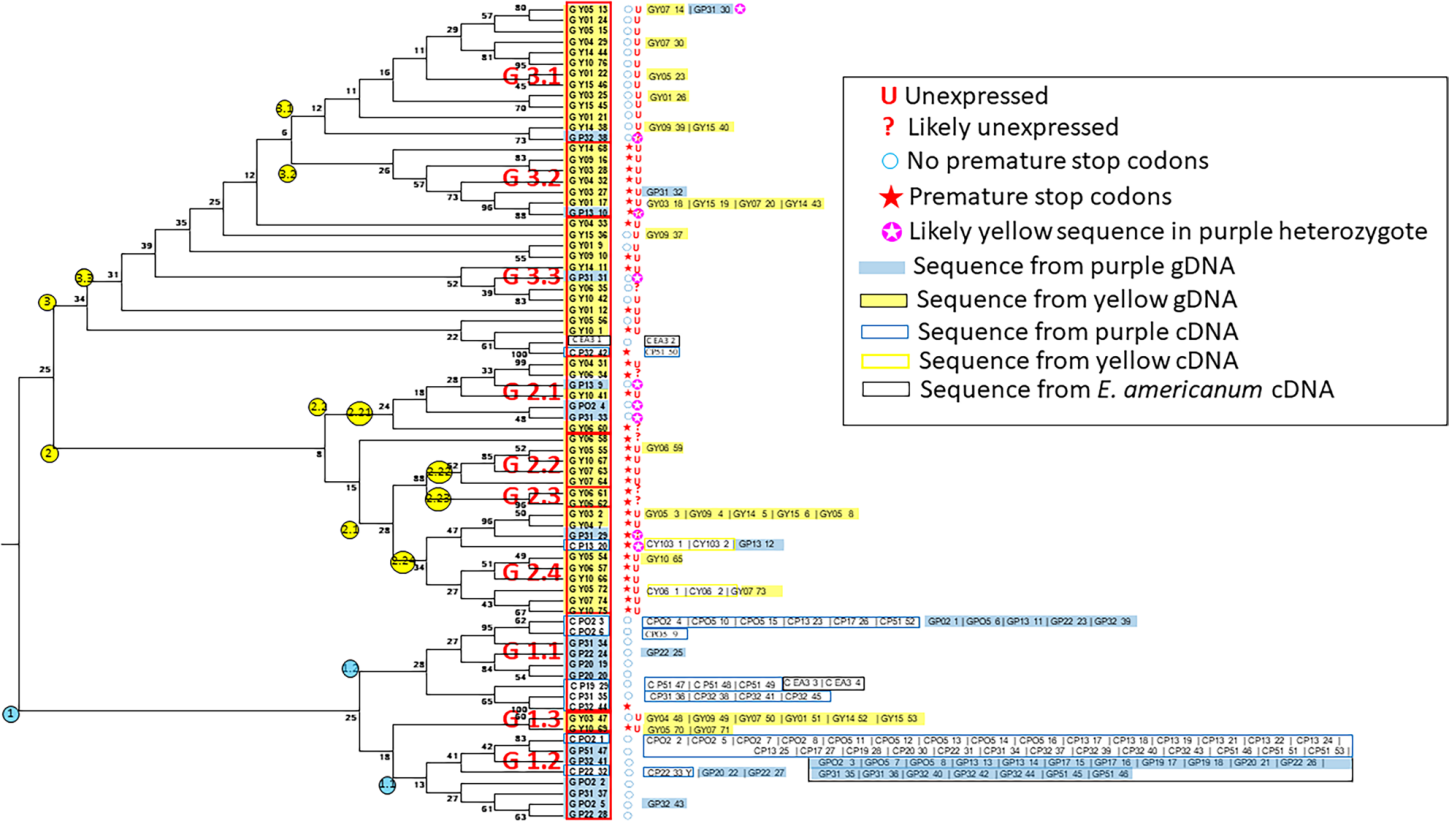
Consensus maximum-likelihood gene tree of *EuMYB3* paralogs. Tree is based on one exemplar from each equivalence group, indicated by the sequence ID’s at the tips of the tree. Sequence ID code: G = gDNA sequence; C = cDNA sequence; PXX = plant ID of purple individual XX; YXX = plant ID of yellow individual XX; EA3 = a purple/red-anthered *Erythronium americanum* individual*.* Last number is a unique number for each sequence. Numbers at nodes indicate percent of bootstrap trees that include the node. Yellow and blue circles indicate clade ID. Sequence Groups (see text) are indicated by red boxes and red group numbers to their left of the boxes. Open circles: sequence contains no premature stop codons. Red stars: sequence contains premature stop codons. Red “U”: sequence not expressed in anther tissue. Red “?”: Likely not expressed in anther tissue because although individual expresses one paralog, it is not indicated copy. Boxes with sequence ID’s to right of tree: sequences from same equivalence group as exemplar. Color codes for sequence ID’s: solid yellow: gDNA from yellow individuals; yellow outline: cDNA from yellow individuals; solid blue: gDNA from purple individuals; blue outline: cDNA from purple individuals. White star in pink circle: likely a “yellow” sequence from a heterozygous purple individual.

To make sense of this paralog diversity, we constructed a maximum-likelihood consensus gene tree using one representative of each equivalence class (Fig. 4). Although bootstrap support is not high, especially in the interior nodes, there is a clear pattern: in general, sequences from purple individuals fall into two clades (Groups 1.1 and 1.2 in Fig. 4), while sequences from yellow individuals fall into two different major clades (2 and 3 in Fig. 4) and one minor clade (Group 1.3 in Fig. 4). The two yellow major clades can be further divided to subclades: Groups 2.1, 2.2, 2.3, 2.4, 3.1, 3.2 and 3.3 (Fig. 4). Moreover, the multiple sequences from a given individual are generally distributed among different groups (Supplementary Table S4). For example, for the individual Y06, 2 copies were located in each of Groups 2.1, 2.2, and 2.3, and 1 copy in each of Groups 2.4 and 3.3. Of the 100 possible Group × Individual combinations (10 Groups × 10 individuals/Group), only 3 had 3 sequences; the remainder had 2 or fewer, as would be expected if each Group represents a different paralog. Those with 3 sequences likely represent minor classification errors. We tentatively conclude that the identified Groups correspond to different paralogs.

Most sequences from purple plants fall into either of Groups 1.1 and 1.2, with sequences falling only sporadically in the other Groups. This pattern is consistent with Groups 1.1 and 1.2 representing copies potentially activating anthocyanin enzyme-coding genes. Based on the number of yellow plants sampled in the population (18.31%, Supplementary Table S1), the frequency of the yellow allele was estimated to be ~ 0.4, and thus, approximately half of the purple plants are expected to be heterozygotes, assuming a Hardy–Weinberg equilibrium. Consequently, we would expect to see some copies from some purple plants in sequence clades that contain primarily sequences from yellow plants. This pattern is evident in Fig. 4. The 13 sequences from purple plants in Groups 2.1, 2.4, 3.1, 3.2, and 3.3 are recovered from only 5 of the 10 purple plants in our study (P02, P13, P31, P32, and P51). If these represent “yellow” sequences from heterozygotes, then purple and yellow sequences actually fall completely into separate Groups.

We used a permutation test to determine whether the proportions of sequences from purple and yellow plants were differentially distributed among “purple” Groups (Groups 1.1 and 1.2) and “yellow” Groups (all remaining groups). For this analysis, we only used one sequence from a given equivalence class for an individual. Among the observed sequences, all 80 yellow sequences fell into “yellow” Groups; among the 100 purple sequences, 87 were in “purple” Groups, and only 13 in “yellow” Groups. This yielded an observed contingency correlation of Ф_obs_ = 0.8713. A permutation test indicated that 0 out of 10,000 permutations yielded a value of Ф as large as the observed value, suggesting that the probability, *P*, of the null hypothesis is < 0.0001. Using *P* = 0.0001, the 99.9% confidence interval for *P* is (− 0.000426, 0.000227), indicating that the deviation from random distribution of sequences among the groups is highly significant.

Two important implications of this result are: (1) the *EuMYB3* paralogs are linked and in strong linkage disequilibrium, and (2) the region containing these paralogs contains the variants responsible for the anther-color polymorphism. These conclusions are based on the following logic: First, in purple plants, the primary functional copy appears to be that represented by Group 1.2. We are not certain which yellow group contains sequences orthologous to those of Group 1.2, and we first hypothesize that this is Group 1.3. Then sequences in the other yellow Groups should not represent the causal locus. If they were unlinked to the paralog of Group 1.3, then recombination would produce no association between those groups and anther color. However, there clearly is an association, which means that the assumption that they are unlinked to the Group 1.3 paralog is not correct. A similar argument holds if any of the other paralogs are orthologous to the purple Group 1.2 sequences. Given that the paralogs are linked, the association between color and genotype in this region (as indicated by whether sequences from an individual fall into purple or yellow groups) implies the region contains the anther-color variant.

Although we cannot determine which of the paralogs represented by the yellow Groups is orthologous to the purple Group 1.2 sequences, a number of properties indicated that many of yellow group sequences are non-functional or not expressed (Table 2). First, Groups 2.1, 2.4, 3.1, 3.2 and 3.3 all have significantly higher π_N_ and π_S_ than purple Group 1.2, suggesting that purifying selection on sequences in these groups is substantially relaxed. This is consistent with the relatively high π_N_/π_S_ ratios in these groups (Table 2). Although these ratios are similar to that seen in purple Group 1.2, the small number of bp differences among sequences in that group makes the estimated π_N_/π_S_ value likely unreliable. Consistent with relaxed selection, sequences in Groups 2.2, 2.3, 2.4, and 3.2 have several premature stop codons (Supplementary Dataset S1), as do some sequences in Group 3.3. These results suggest that paralogs in Groups 2.1, 2.2, 2.4, and 3.2 are pseudogenes.

**Table 2:** Characteristics of sequences in the different Groups defined in Fig. 4.

Yellow group	Expressed	Premature stop codons	π_N_			π_S_			π_N_/π_S_		
			Purple	Yellow	*P*	Purple	Yellow	*P*	Purple	Yellow	*P*
1.3	No	Some	0.0028	0.0034	0.9	0.00042	0.00068	0.772	1.289	1.408	0.998
2.1	No*	Yes	0.0028	0.0222	0.002	0.00042	0.0111	< 0.002	1.289	0.582	0.97
2.2	No	Yes	0.0028	0.0029	0.462	0.00042	N/A	N/A	1.289	N/A	N/A
2.4	No	Yes	0.0028	0.0092	0.006	0.00042	0.0022	0.024	1.289	1.289	0.44
3.1	No	No	0.0028	0.0104	< 0.002	0.00042	0.0105	< 0.002	1.289	0.741	0.97
3.2	No	Yes	0.0028	0.0103	< 0.002	0.00042	0.0103	< 0.002	1.289	0.409	0.95
3.3	No	Some	0.0028	0.0332	< 0.002	0.00042	0.012	< 0.002	1.289	0.854	0.98

“Expressed” indicates whether sequences are expressed in yellow individuals. The asterisk indicates some sequences in that group are not expressed (see Fig. 4 and text for explanation). “Premature stop codons” indicates whether sequences have premature stop codons. “Some” indicates only some of the sequences in a given group have premature stop codons (see Fig. 4). π_N_, π_S_, and π_N_/π_S_ are indicated separately for purple and yellow sequences from a given group. *P* is significance of difference between these measures of sequence diversity.

Of the remaining yellow Groups, 1.3, 3.1 and 3.3 appear to be unexpressed. This inference is based on lack of expression of any of the paralogs in all yellow individuals except Y06 and Y103 (Fig. 3). The sequences expressed in these two individuals are located in Group 2.4 and have 10 and 1 premature stop codons each and are thus likely non-functional. It thus appears that all yellow groups contain sequences that are either not expressed, not functional or both. Regardless of which yellow group is orthologous with the functional purple sequences in Group 1.2, this pattern indicates that lack of a functional and expressed copy of *EuMYB3* means that the downstream genes of the anthocyain biosynthetic pathway are not expressed in plants with yellow anthers, and hence no pigment is produced.

### Balancing selection is not detectable on *EuMYB3* loci

Although we do not know which yellow paralog corresponds to the causal locus for anthocyanin pigmentation, we can test each paralogous group to determine whether, in combination with purple Group 1.2, there is any evidence for selection. We first assessed whether Tajima’s D statistic was positive, which is expected if there is balancing selection. For all yellow groups, D was < 0 (Table 3), providing no evidence for balancing selection. A test was not performed for Group 2.3 because it contains only two sequences.

**Table 3:** Tests for balancing selection for combinations of sequences in the purple Group 1.2 and sequences in the assigned yellow group.

Yellow group	Yellow sample size	Tajima’s D	Recomb. rate	Tolerance					
				0.01		0.05		0.1	
				Fst	N diff	Fst	N diff	Fst	N diff
1.3	10	− 0.796	0	0.381	0.159	0.353	0.145	0.315	0.142
			2	0.366	0.115	0.328	0.117	0.312	0.099
			4	0.308	0.063	0.296	0.066	0.319	0.068
			8	0.366	0.023*	0.35	0.030*	0.292	0.030*
2.1	7	− 2.244	0	0.362	1.000	0.337	1.000	0.351	1.000
			2	0.361	1.000	0.376	1.000	0.337	1.000
			4	0.421	1.000	0.347	1.000	0.323	1.000
			8	0.350	1.000	0.339	1.000	0.303	1.000
2.2	8	− 2.218	0	0.309	0.750	0.313	0.685	0.284	0.610
			2	0.295	0.633	0.300	0.599	0.272	0.543
			4	0.302	0.516	0.319	0.534	0.291	0.494
			8	0.233	0.477	0.267	0.488	0.237	0.446
2.4	13	− 1.627	0	0.197	0.202	0.229	0.214	0.223	0.229
			2	0.231	0.151	0.233	0.150	0.211	0.161
			4	0.244	0.151	0.246	0.135	0.208	0.118
			8	0.193	0.104	0.197	0.094	0.183	0.097
3.1	12	− 2.418	0	0.336	0.394	0.334	0.407	0.297	0.386
			2	0.357	0.331	0.314	0.324	0.284	0.317
			4	0.321	0.294	0.305	0.301	0.28	0.294
			8	0.339	0.267	0.305	0.266	0.289	0.266
3.2	7	− 1.456	0	0.209	0.097	0.198	0.117	0.154	0.097
			2	0.173	0.043*	0.164	0.084	0.158	0.075
			4	0.124	0.046*	0.118	0.049*	0.112	0.072
			8	0.153	0.046*	0.114	0.038*	0.100	0.035*
3.3	9	− 1.665	0	0.236	0.120	0.251	0.149	0.226	0.155
			2	0.202	0.067	0.186	0.076	0.193	0.092
			4	0.201	0.077	0.197	0.069	0.175	0.076
			8	0.138	0.017*	0.177	0.015*	0.151	0.027*

Values for coalescence test are the proportion of bootstrap replicates that are greater than observed measure of divergence (Fst and N diff).

*N diff* number of fixed silent differences between purple and yellow allele classes. Tolerance is parameterized at three levels: 0.01, 0.05, and 0.1.

*Indicates *P* < 0.05.

The coalescent test for balancing selection provides little evidence for the operation of balancing selection. A total of 168 tests were performed (2 divergence measures × 7 Groups × 3 tolerance levels × 4 recombination levels = 168). Of these, 12 were nominally significant at *P* < 0.05 and none were nominally significant at *P* < 0.01 (Table 3). The probability of obtaining 10 significant results (at *P* < 0.05) by chance out of 144 tests is 0.185, indicating that the nominally significant results are likely false positives. Based on the results from the Tajima’s D and coalescent tests, we conclude that our data provides little evidence of historical balancing selection on the copy of *EuMYB3* responsible for the anther-color polymorphism.

## Discussion

One goal of this study is to determine the type of genetic changes that are responsible for the anther color difference in *E*. *umbilicatum*. This purple/yellow anther-color polymorphism results from the presence/absence of cyanidin-derived anthocyanins. Absence of anthocyanins in the yellow anthers is correlated with downregulation of three ABP enzyme-coding genes, *EuDfr*, *EuAns* and *EuUf3gt*.

Our data clearly rule out the possibility that functional mutations in these ABP enzyme-coding genes cause lack of pigmentation in the yellow anthers because the same copies of the enzyme-coding genes are expressed in the anther and leaf tissues of *E*. *umbilicatum*, and cyanidin-derived anthocyanins are produced in the leaves (Fig. 1), implying functionality of all three enzymes. Another possibility is that *cis*-regulatory mutations cause downregulation of *EuDfr*, *EuAns* and *EuUf3gt*. This situation would require that all of 10 yellow individuals carried a regulatory mutation in each of the three genes. Although possible, the likelihood of this occurring in our samples, is extremely low (6.21 × 10^–29^, see Supplementary Methods S1 for the details of likelihood estimation), implying that coordinate downregulation of the three genes is more likely explained by a mutation occurring in a common regulator. Such a regulator could be a repressor. Typical anthocyanin repressors in plants are *R3-MYBs*^27^, but we found no such genes in our transcriptiomes, suggesting that variation at a repressor is an unlikely explanation. By contrast, we detected downregulation of two transcription activators, *EubHLH2* and *EuMYB3 *in yellow anthers.

While either or both of these genes may control the color polymorphism, we focused on characterizing *EuMYB3*, specifically asking whether genotype at this locus is correlated with anther color. Although *EuMYB3* turned out to be a complex “locus” with several paralogs, we found a strong association between anther color and which *EuMYB3* sequences were present. In particular, most sequences from purple plants fell into two Groups (paralogs), while no yellow plants had sequences in those Groups. By contrast, all yellow sequences fell into the remaining “yellow” Groups. Although a few sequences from purple plants grouped with the “yellow” Groups, these can reasonably be interpreted as “yellow” sequences from purple heterozygotes. Thus, sequence genotypes are strongly associated with color phenotypes, implicating one of the *EuMYB3* paralogs as the causal locus. We note also that the assortment of purple and yellow sequences into different sequence groups (generally corresponding to clades) implies the paralogs are found in the same genomic region and are linked.

It seems likely that the sequences in Group 1.2, all from purple plants, represent the main *R2R3-MYB* activating the enzyme-coding genes, although the paralog corresponding to Group 1.1 may also contribute. From our data we cannot determine which yellow paralogs correspond to these two purple paralogs. Yellow Group 1.3 perhaps seems likely to correspond to the purple Group 1.2 paralog because these two groups are sister clades. However, yellow paralogs in other clades appear to be subject to reduced or absent purifying selection, as evidenced by both their substantially elevated π_N_ and π_S_ values and their high π_N_/π_S_ ratios. This accelerated accumulation of mutations due to relaxed selection would tend to reduce their sequence similarity to the purple sequences even if they represent the same paralogs as the purple paralogs.

Nevertheless, regardless of which paralog a yellow sequence belongs to, it appears to be either non-functional due to the accumulation of premature stop codons or greatly downregulated. Both of these possibilities can explain lack of expression of the downstream ABP genes *EuDfr*, *EuAns* and *EuUf3gt*. One possible caveat to this conclusion is that two yellow individuals express a copy of *EuMYB3*. However, these copies have premature stop codons which likely render them non-functional.

Because we did not examine the ABP transcription factor *EubHLH2*, we cannot rule out the possibility that its reduced expression in yellow-anthered individuals is contributing to the absence of pigmentation. However, two considerations mitigate against it being the primary gene responsible for the color polymorphism. One is that in *Arabidopsis*, *Petunia*, and Asiatic hybrid lily (*Lilium* spp.), the ABP *R2R3-MYB* is known to activate ABP *bHLH* genes, possibly by binding between the two proteins^28–31^. Second, the correlation between *EuMYB3* genotype and color phenotype is very high (0.87), leaving little scope for another locus to have a large effect on anther color. For these reasons, we believe it is reasonable to conclude the *EuMYB3* has the largest effect on anther color.

Based on Tajima’s D and coalescent tests, we found no evidence of long-term balancing selection acting on *EuMYB3* for any of its paralogs, suggesting that balancing selection has not acted historically on the anther-color polymorphism. Several factors, however, complicate this interpretation. First, although *EuMYB3* appears to account for most of the variation in anther color, we cannot rule out minor contributions from other genes such as *EubHLH2*. If multiple genes contribute to variation in a discrete phenotype, under some circumstances phenotypic balancing selection may not preserve variation at all of those genes. We cannot definitively rule out this possibility, but we note that it seems intuitively unlikely that a major locus responsible for a balanced phenotypic polymorphism will not itself experience balancing selection.

Another complicating factor is that balancing selection may have been operating on anther color for only a short time, so that there has been insufficient time for signatures of balancing selection to increase to detectable level. Both of our measures of allele-class divergence rely on prolonging the coalescence time for those allele-classes so that divergent mutations can accumulate. This is, of course, a limitation of any study that attempts to detect balancing selection by identifying signatures of selection, and can only be overcome by actually quantifying selection in nature.

A final complicating factor is that we simply may have lacked sufficient power to detect signatures of selection. This seems unlikely for Tajima’s test, since D values were negative and large, but may be an issue for the coalescence test. Overcoming this limitation would require larger sample sizes than were used in this study. Additionally, the negative D values could be due to recent population expansion, which could possibly mask any balancing selection occurring. Despite these caveats, we tentatively conclude that balancing selection on this conspicuous polymorphism is not occurring, and that the color variation represents a transient state in which one color or the other will be fixed, either by drift or selection.

This absence of selection may not be surprising. There is little evidence that anther color variation affects interactions with pollinators that would generate any sort of negative frequency-dependent selection. One study on the polymorphism in *E. americanum* found no effects of anther color on herbivory, tolerance of UV-B radiation, or on siring success^11^. While pollinators exhibited different site-specific preferences, a balance between divergent selection and migration could generate balancing selection that maintains variation within sites, but only if there is substantial pollen or seed flow between sites. Because *Erythronium* seeds are dispersed primarily by ants, dispersal appears to be limited largely to a few meters^32–34^. Pollinators are primarily flies and solitary bees, which cannot disperse pollen far, and sometimes honeybees^11,13^, which are capable of longer-distance dispersal. Whether such dispersal is sufficient to generate a selection-mutation balance is unclear and needs further examination.

A final conclusion from our analysis regards the evolution of anthocyanin regulation. Coordinated expression of multiple enzyme-coding genes is common in the anthocyanin transcriptional regulation. In general, the pathway can be divided into two subsets: early biosynthetic genes (EBGs) and late biosynthetic genes (LBGs), although the genes grouped into EBGs or LBGs vary among species^18,20^. The genes within a single subset are regulated coordinately usually by an R2R3-MYB and/or a bHLH transcription factor. In eudicots, EBGs and LBGs are usually regulated separately by different sets of transcription factors^20,35–37^, although this does not appear to be true in *Ipomoea purpurea*^38^. By contrast, in monocots, studies on maize and Asiatic hybrid lily have revealed that both EBGs and LBGs (i.e., the entire enzyme-coding genes) are regulated by the same set of transcription factors^19,39^. However, in the white hybrid of the orchid *Dendrobium* spp., only *F3h*, *Dfr*, and *Ans* are found to be downregulated coordinately^40^. Our findings also show that EBGs and LBGs are controlled separately in some monocots. In *E. umbilicatum*, the expression change in *EuDfr*, *EuAns* and *EuUf3gt* occurs simultaneously, but this change is not shared with *EuChs*, *EuChi*, *EuF3h* and *EuF3’h*, which suggests that *EuDfr*, *EuAns* and *EuUf3gt* belong to LBGs and are regulated coordinately. Interestingly, although this transcriptional regulation pattern is different from the pattern found in another species in Liliaceae and other monocots, it matches the pattern in some eudicot species, such as *Petunia*^41^. Our findings thus provide an additional example that the anthocyanin regulatory network is evolutionarily very labile^17,42^.

## Methods

We collected *E. umbilicatum* anthers and leaves, and examined biochemical and genetic basis of anther colors. All methods describe below were carried out in accordance with relevant guidelines and regulations.

### Sample collection

Immediately before anther dehiscence, the nodding flower buds of *E. umbilicatum* were collected in March 2015 and March 2016 in the Oosting Natural Area (35° 58ʹ 48.5ʹʹ N, 79° 03ʹ 54.7ʹʹ W) of Duke Forest in Orange County, North Carolina, USA. This sampling method ensured that all buds were at the same developmental stage. These buds were then brought to the lab. After petals and sepals were removed, the anthers were scored as purple or yellow, and then stored at − 80 °C until use.

### Characterization of anthocyanidins in anthers and leaves

Pigments in the *E. umbilicatum* anthers were characterized using high performance liquid chromatography (HPLC). One purple and one yellow anther sample were prepared separately by pooling 12 anthers (approximately 60 mg) collected from six purple plants, and 12 anthers from six yellow plants. Extraction and identification of anthocyanidins, the aglycone precursors of anthocyanins, were conducted following the Methods described in Supporting Information Methods S1 in Ref.^17^.

Anthocyanidins isolated from the anthers and leaves of the same *E. umbilicatum* plants (six anthers and one leave from individual plants) were also characterized using thin layer chromatography (TLC). The extracts and standards were run on a cellulose plate (Sigma-Aldrich, St. Louis, MO, USA) in the Forestal solvent (glacial acetic acid: conc. HCl: water = 30:3:10, v/v/v) until the standards were separated and before the solvent front ran to the end of the plate. Standards of cyanidin, pelargonidin, malvidin (Indofine Chemical Company, Hillsborough, NJ, USA) and delphinidin (Polyphenols Laboratories, Sandnes, Norway) were included in the run.

### Transcriptomics

We performed RNA sequencing on purple and yellow anthers*.* Total RNA was extracted from 1 to 2 anthers from each plant using Spectrum Plant Total RNA Kit (Sigma-Aldrich). One library of the purple morph and one library of the yellow morph were prepared individually by pooling equal amounts of RNA from 50 purple and 50 yellow plants. RNA quality examination, library construction, barcoding, and sequencing were performed following Supporting Information Methods S2 in Ref.^17^.

Bioinformatic analyses, including trimming of raw reads, assembling of transcripts, identification of anthocyanin gene candidates and estimation of gene expression, were conducted as described in Ref.^17^. The sequences of anthocyanin genes from *Arabidopsis*, *Petunia*, *Lilium*, and *Tulipa* and the sequences of *R3-MYB* repressors (Supplementary Table S2) were used as queries in BLAST searches.

### Cloning of the ABP enzyme-coding genes

To determine whether the same copies of the ABP enzyme-coding genes were expressed in the anther and leaf tissues of *E. umbilicatum*, the full-length coding regions of *EuChs*, *EuChi*, *EuF3h*, *EuF3ʹh*, *EuDfr*, *EuAns*, and *EuUf3gt* were amplified using anther cDNA and leaf cDNA as templates. Total RNA was extracted from the anthers and leaves of one purple *E. umbilicatum* plant as described above. Before making cDNA, RNA was first treated with RQ1 RNase-Free DNase (Promega, Madison, WI, USA) to remove residual genomic DNA (gDNA). cDNA was synthesized as described in Supporting Information Methods S4 in Ref.^17^. PCR primers (Supplementary Table S3) were designed using Primer3 (https://primer3.ut.ee/) based on the sequences retrieved from the transcriptome assemblies. Details of amplification, cloning, and sequencing were described in Supporting Information Methods S5 in Ref.^17^.

### Cloning of the ABP-associated transcription factors

We also amplified and cloned the full- or partial-length coding regions of the ABP-associated transcription factors identified from anthers (*EuMYB3*, *EubHLH2*, *EuWDR1* and *EuWDR2*). The cDNA samples were prepared with anther RNA extracted from one purple plant and one yellow plant. *EubHLH1* was not included because, despite several attempts, the amplification of *EubHLH1* has never been successful. PCR primers (Supplementary Table S3) were designed based on the sequences retrieved from the transcriptome assemblies, except for *EubHLH2*. Because we only retrieved a short fragment of *EubHLH2* from the transcriptome, the forward primer to amplify this gene was designed based on a conserved region of *LhbHLH2* (GenBank accession number: AB222076, from *Lilium* spp.) and *TfbHLH2* (GenBank accession number: KF924736, from *Tulipa fosteriana*), and the reverse primer was designed with the aid of 3ʹ RACE (FirstChoice RLM-RACE Kit, Ambion, Austin, TX, USA).

To evaluate whether *EuMYB3*, *EubHLH1* and *EubHLH2* are homologs to the known anthocyanin regulators, we constructed a phylogenetic tree of EuMYB3 and the related R2R3-MYB proteins, and also a phylogenetic tree of EubHLHs and the related bHLH proteins. The detailed methods are provided in Supplementary Methods S2.

### Quantification of gene expression in purple and yellow anthers

Based on the FPKM values (the number of RNAseq fragments per kilobase of transcript effective length per million fragments mapped to all transcripts) from our transcriptome data, we selected five enzyme-coding genes (*EuChs*, *EuF3h*, *EuDfr*, *EuAns* and *EuUf3gt*) and three transcription factors (*EubHLH2*, *EuWDR1* and *EuWDR2*) to further analyze their expression in the purple and yellow anthers using quantitative real-time PCR (qPCR). *EuMYB3* was not included because multiple gene copies were detected, and high sequence similarity among these paralogs made it impossible to design paralog-specific primers (see “Results” section). The anther cDNA was synthesized as described above and then diluted to 2.5 ng/μL for qPCR. The qPCR primers (Supplementary Table S3) were designed to amplify 80–150 bp fragments of the selected genes and *EF1α* (*elongation factor 1-alpha*, serving as a reference gene). The detailed protocol is described in Supporting Information Methods S7 in Ref.^17^. The qPCR assays were conducted with ten biological replicates for each color morph and two technical replicates for each sample. One cDNA sample from a purple individual was arbitrarily chosen as a control sample and was included in each run. The relative expression ratios of target genes were normalized with the expression of *EF1α*, using the Eq. (1) in Ref.^43^. The detailed calculation is described in Supplementary Methods S3. The relative expression levels were then calculated as the logarithm of the ratios.

### Semi-quantification of gene expression

Our FPKM and qPCR results show undetectable expression levels of *EuDfr*, *EuAns*, *EuUf3gt* and *EubHLH2* in the yellow anthers. However, given that (i) the same copies of enzyme-coding genes were expressed in the anthers and leaves of the purple *E. umblicatum* and (ii) leaves of the yellow plants contain anthocyanins (see “Results” section), we expected that *EuDfr*, *EuAns* and *EuUf3gt* should be expressed in the leaves of the yellow individuals. In addition, because *bHLH* genes often have broader expression domains^44,45^, we presumed that *EubHLH2* was expressed in the leaves of the yellow plants as well. By contrast, anthocyanin-regulating *R2R3-MYB* genes often have specific expression domain, we predicted that *EuMYB3* would be expressed in the anthers only.

To compare the expression levels of *EuDfr*, *EuAns*, *EuUf3gt*, *EubHLH2* and *EuMYB3* in the anther and leaf tissues, we used semi-quantitative PCR with three biological replicates for each color morph. The PCR reactions were conducted with *Taq* DNA Polymerase (New England BioLabs, Ipswich, MA, USA) using anther cDNA or leaf cDNA as templates. For *EuDfr*, *EuAns*, *EuUf3gt*, and *EubHLH2*, the same qPCR primers (Supplementary Table S3) were used, while for *EuMYB3*, we used primers MYB3-1F and MYB3-Q2R (Supplementary Table S3) to amplify the full-length of *EuMYB3*. PCR products were visualized on 2% agarose gels (1.2% agarose gels for *EuMYB3*). *EF1α* was included as a positive control.

Since the presence of *EuMYB3* paralogs made it not suitable to quantify *EuMYB3* expression using qPCR, we again employed semi-quantitative PCR to detect *EuMYB3* expression differences between purple and yellow anthers. The PCR reactions were conducted as described above, using anther cDNA (*N* = 10 for each color morph) as templates.

### Analysis of *EuMYB3* copies in purple and yellow anthers

As documented in the Results section, multiple paralogs of *EuMYB3* exist within *E. umbilicatum*. To identify these paralogs, and to quantify sequence variation within and between paralogs, we cloned the full-length *EuMYB3* that were amplified with the primers MYB3-1F and MYB3-Q2R (Supplementary Table S3). Templates used in PCR reactions are gDNA of 10 purple (Plant IDs: P02, P05, P13, P17, P19, P20, P22, P31, P32, P51) and 10 yellow individuals (Plant IDs: Y01, Y03, Y04, Y05, Y06, Y07, Y09, Y10, Y14, Y15), and anther cDNA of the same 10 purple and 2 yellow individuals (Plant IDs: Y06 and Y103). Few cDNA samples of yellow plants were included because *EuMYB3* is rarely expressed in the yellow anthers (see “Results” section). For anther cDNA samples of the five purple plants, P02, P05, P13, P32, and P51, cloning was repeated three times with separately amplified PCR products, and at least in one of these three replicates, cDNA synthesized in different reactions was used. We also amplified this gene from anther cDNA of one *E. americanum* sample (Plant ID: EA3; purple/red-anthered). The details of cloning were described in Supporting Information Methods S5 in Ref.^17^. We then obtained 184 sequences (GenBank accession numbers: OK648453–OK648456, OP963196–OP963318 and OP963321–OP963377) from 426 colonies. Of these sequences, 47 and 76 were respectively obtained from gDNA of purple and yellow individuals, and 53, 4, and 4 were respectively obtained from anther cDNA of purple, yellow, and an *E*. *americanum* plants.

To assign sequences to different paralogs, we first collapsed the dataset by assigning each sequence to an “equivalence class” using a custom APL script written by MDR. Sequences in each equivalence class differed at no more than 1 bp in the coding region. This assignment was done to account for possible sequencing errors. We then used one sequence from each equivalence class, which reduced the dataset to 76 sequences, to construct a maximum-likelihood gene tree using MEGA^46^ (Version 10.1.8) with the following parameters: General Time reversible Model with Gamma distributed rates with 5 categories. Uncertainty in the tree was assessed by conducting 500 bootstrap replicates. As described in the Results section, the resulting gene tree had several candidate sequence groupings that appear to correspond to different paralogs.

Although we identified the grouping of purple sequences that correspond to *EuMYB3*, we cannot identify which grouping of yellow sequences is orthologous to *EuMYB3*. Consequently, to assess whether patterns of variation differed between purple and yellow copies of *EuMYB3*, we compared each yellow grouping to the purple *EuMYB3*. We identified synonymous and non-synonymous SNPs for each comparison and calculated π_N_, π_S_, and π_N_/π_S_. To test whether these parameters differed between the purple and yellow sequences, we performed a bootstrap analysis with 500 replicates. These analyses were performed using custom APL scripts written by MDR.

For each pair of groupings, we also conducted a coalescent analysis to test whether divergence between purple and yellow sequences is greater than expected under neutral evolution. The causal locus (SNP) defines two allele classes corresponding to each allele. Under neutrality and in the absence of recombination, the region surrounding the causal locus is expected to diverge between the two allele classes because they independently accumulate mutations at different position, either through genetic drift or natural selection. Because balancing selection prolongs the time available for accumulation of such mutation, divergence is expected to be greater than under neutrality.

This difference suggests the following coalescence test for balancing selection, kindly suggested by Matthew Hahn (Indiana University). Separate analyses were performed comparing purple Group 1.2 to different yellow Groups. For each comparison, the number of sequences modeled was equal to the number of samples in the sequence set for the Groups being compared. A Group’s sequence set included at least one sequence from each individual with a sequence in the Group. For individuals that had two distinct sequences in the Group, we included both samples. The number of sequences in the sample set for each yellow Group is listed in Table 3. The number of samples in the purple Group 1.2 sequence set was 14. Modeling specified a fixed number of SNPs, which was the number of SNPs in the sequence sets for the compared groups.

We first estimated observed divergence based on three different divergence measures calculated from the observed sequences: (1) Average Fst; (2) E, the Euclidian distance between the purple and yellow sequences; and (3) FD, the number of SNPs with fixed differences between the two allele classes. We then used the program ms^47^ to generate 1000 replicate coalescent samples using the same number of SNPs plus 1 (to represent the causal SNP) and sample sequence numbers equal to the sizes of the two Group sample sets, constraining the number of SNPs to be equal to the observed number. For each Group comparison, we ran separate analyses using different recombination rates: none, 2 cM/Mb, 4 cM/Mb and 8 cM/Mb. Because the recombination rate in *E. umbilicatum* is not known, we chose these values to span typical recombination rates in plants (mean = 1.85 cM/Mb^48^). Simulations assumed a gene size of 1000 bp. Using APL scripts (written by MDR), for each coalescent sample we identified a SNP with the same frequency of the yellow allele in our sampled population to represent the causal SNP. Because there will very rarely be a SNP in the coalescent sample that has a frequency exactly equal to that of yellow allele in the real population, we accepted a SNP from the simulated sample if its frequency difference from the actual frequency was less than a certain tolerance level. Three different tolerance levels were used: 0.01, 0.05 and 0.1.

For each coalescent sample, we used the “causal” SNP to divide the coalescent sample haplotypes into two sets, representing purple and yellow sequences, according to genotype at that SNP. We then calculated Fst, E, and FD for the coalescent sample. Finally, we calculated the proportion of coalescent samples with values of these statistics greater than the observed values. These proportions represent the *P* values of the null hypothesis that the observed values are consistent with neutral divergence.

### Supplementary Information

Supplementary Information.

## Data Availability

The sequences generated in this study were deposited at NCBI under GenBank accession numbers: OK648430–OK648456 and OP963196–OP963377. Raw reads and transcript assemblies of RNA sequencing were deposited at NCBI under BioProject PRJNA905549: Sequence Read Archive (SRA): SRR22414735 and SRR22414736; Transcriptome Shotgun Assembly (TSA): GKDT00000000 and GKDU00000000. The TSA described in this paper is the first version.
