# Supplementary material for: Absence of long-term balancing selection on variation in EuMYB3, an R2R3-MYB gene responsible for the anther-color polymorphism in Erythronium umbilicatum
Source: Sci Rep. 2024 Mar 4;14:5364. doi: 10.1038/s41598-024-56117-y (PMC10912454; doi:10.1038/s41598-024-56117-y)
Supplement: Supplementary file 1 — Supplementary Information. [file 41598_2024_56117_MOESM1_ESM.pdf]

Supplementary information

Article title: Absence of long-term balancing selection on variation in *EuMYB3*, an *R2R3-MYB* gene responsible for the anther-color polymorphism in *Erythronium umbilicatum*

Authors: Rong-Chien Lin, Mark D. Rausher

Supplementary Tables

Table S1 The *Erythronium umbilicatum* anther-color polymorphism in 16 sites in North Carolina, USA
Table S2 The query sequences used in the BLAST searches
Table S3 Primers used in this study
Table S4 Numbers of gDNA sequences of *EuMYB3* in different sequence Groups for purple and yellow individuals

Supplementary Figures

Figure S1 Neighbor-joining phylogenetic tree of R2R3-MYB proteins
Figure S2 Neighbor-joining phylogenetic tree of bHLH proteins
Figure S3 Alignment of anthocyanin-regulating R2R3-MYB proteins
Figures S4-S13 The original, unprocessed gel photographs used in Figure 3. The gels were imaged using the gel imager Bio-Rad Gel Doc XR system. The top and bottom halves of the whole gel were imaged separately due to the UV illumination issue of our gel imager. The photographs were then cropped to create the panels in Figure 3 using POWERPOINT and ILLUSTRATOR.

Supplementary Methods

Methods S1 Likelihood estimation of joint *cis*-regulatory mutations leading to triple downregulation
Methods S2 Phylogenetic analyses of R2R3-MYB and bHLH proteins
Methods S3 Calculation of relative expression in qPCR analyses

Supplementary Dataset

Dataset S1 EuMYB3 amino-acid sequences with premature stop codons (indicated by *)
Dataset S2 The TIFF files of Supplementary Figures S4-S13, which were directly exported from the Bio-Rad Gel Doc XR system (provided in a separate zip file). Each TIFF file is named with "(FigS*n*)", where "*n*" represents the figure number.

Table S1 The *Erythronium umbilicatum* anther-color polymorphism in 16 sites in North Carolina, USA. These 16 sites are distributed along three main rivers/creeks: Eno River, New Hope Creek/Old Field Creek and Morgan Creek. We conducted an anther-color survey during the flowering season in 2017. In each site, we set up 2 m x 2 m plots that were 6 m apart from each other. Within each plot, four 1 m x 1 m quadrats were laid out. We tallied the anther color for all plants in each quadrat, and counted at least 200 plants in each site. The geographical coordinates of each site, the number of flowers surveyed, the number of flowers having purple or yellow anthers, and the percentage of yellow-anthered flowers in each site are shown.

	Location			Anther color		
Site	Latitude	Longitude	No. of flowers surveyed	Purple	Yellow	% of yellow-anthered flowers
Eno. Old Oxford	36°04’16.8”N	78°51’32.5”W	301	287	14	4.65
Eno. Penny’s Bend	36°04’30.6”N	78°52’05.3”W	292	278	14	4.79
NCBG. Stillhouse Bottom	35°53’06.4”N	79°02’49.9”W	210	198	12	5.71
NCBG. Morgan Creek	35°53’27.3”N	79°02’28.5”W	228	199	29	12.72
NCBG. Piedmont Nature Trails	35°53’49.7”N	79°02’01.4”W	266	256	10	3.76
Duke Forest. Oosting	35°58’48.5”N	79°03’54.7”W	213	174	39	18.31
Duke Forest. Gate 23	35°59’32.1”N	79°02’33.6”W	227	209	18	7.93
Duke Forest. Gate 24	35°59’03.8”N	79°02’03.2”W	218	194	24	11.01
Duke Forest. Gate 26	35°59’03.8”N	79°00’56.9”W	268	244	24	8.96
Duke Forest. Gate 27	35°58’50.5”N	79°00’06.0”W	230	201	29	12.61
Eno. Pleasant Green	36°02’30.4”N	79°00’39.7”W	247	217	30	12.15
Eno. Cabe Lands	36°02’36.6”N	78°59’32.6”W	226	209	17	7.52
Eno. Eno Trace Trail	36°04’26.1”N	79°00’27.6”W	256	205	51	19.92
Eno. Pump Station	36°03’40.5”N	78°58’16.7”W	220	196	24	10.91
Eno. Fish Dam	36°04’02.2”N	78°57’19.6”W	235	226	9	3.83
Eno. Cox Mountain Trail	36°04’57.4”N	79°01’17.1”W	224	182	42	18.75

Table S2 The query sequences used in the BLAST searches. The sequences of *Arabidopsis thaliana* were retrieved from TAIR (https://www.arabidopsis.org/). Other sequences were retrieved from GenBank.

Accession number	Species	Gene
AT5G13930	*Arabidopsis thaliana*	*Chs*
AT3G55120	*Arabidopsis thaliana*	*Chi*
AT3G51240	*Arabidopsis thaliana*	*F3h*
AT5G07990	*Arabidopsis thaliana*	*F3'h*
AT5G42800	*Arabidopsis thaliana*	*Dfr*
AT4G22880	*Arabidopsis thaliana*	*Ans*
AT5G17050	*Arabidopsis thaliana*	*Uf3gt*
AT1G56650	*Arabidopsis thaliana*	*R2R3-MYB*; *AtMYB075*
AT1G66370	*Arabidopsis thaliana*	*R2R3-MYB*; *AtMYB113*
AT1G66380	*Arabidopsis thaliana*	*R2R3-MYB*; *AtMYB114*
AT1G66390	*Arabidopsis thaliana*	*R2R3-MYB*; *AtMYB090*
AT4G09820	*Arabidopsis thaliana*	*bHLH*; *AtTT8*
AT1G63650	*Arabidopsis thaliana*	*bHLH*; *AtEGL1*
AT5G24520	*Arabidopsis thaliana*	*WDR*; *AtTTG1*
AF146702	*Petunia x hybrida*	*R2R3-MYB*; *AN2*
HQ428105	*Petunia x hybrida*	*R2R3-MYB*; *AN4*
AF260919	*Petunia x hybrida*	*bHLH*; *AN1*
U94748	*Petunia x hybrida*	*WDR*; *AN11*
AB534587	*Lilium* hybrid division I	*R2R3-MYB*; *LhMYB6*
AB222075	*Lilium* hybrid division I	*bHLH*; *LhbHLH1*
AB222076	*Lilium* hybrid division I	*bHLH*; *LhbHLH2*
KC261503	*Tulipa fosteriana*	*Chs*
KC261502	*Tulipa fosteriana*	*Chi*
KC261504	*Tulipa fosteriana*	*F3h*
KC256779	*Tulipa fosteriana*	*F3'h*
KC261505	*Tulipa fosteriana*	*F3'h*
KC261506	*Tulipa fosteriana*	*Dfr*
KC261507	*Tulipa fosteriana*	*Ans*
KF792732	*Tulipa fosteriana*	*Uf3gt*
KF990610	*Tulipa fosteriana*	*R2R3-MYB*; *TfMYB3*
KC256778	*Tulipa fosteriana*	*bHLH*; *TfbHLH1*
KF924736	*Tulipa fosteriana*	*bHLH*; *TfbHLH2*
R3-MYB repressor		
AT2G46410	*Arabidopsis thaliana*	*AtCPC*
AT5G53200	*Arabidopsis thaliana*	*AtTRY*
KF985022	*Petunia x hybrida*	*PhMYBx*

JX992854	*Mimulus lewisii*	*MlROI1*
KY658469	*Iochroma cyaneum*	*IcMYBL1*
KY658468	*Iochroma loxense*	*IlMYBL1*
LC429593	*Lilium* hybrid division I	*LhR3MYB1*
LC429594	*Lilium* hybrid division I	*LhR3MYB2*

Table S3 Primers used in this study.

Primers for sequencing the coding regions

Primer name	Primer sequence (5’-3’)
CHS-1F	CAGTTTTTCCCCTTGTCTGTAACA
CHS-1R	AAAAAATAGGATTGTCCCATGC
CHI-1F	CTGTGAAGCTGAGAGAATCCC
CHI-1R	GCAGTTATTTATACTATTACGACG
F3H-3F	CGAGAGAGCTTTCAATCGCA
F3H-2R	CGATAATGTGGCTAAGCATG
F3’H-1F	CACCGCATTCCTCACCCAC
F3’H-3R	ACCCTAGAGCATACTATCCCCA
DFR-2F	CTCAGAGAAACAGAGAGAGAGGAAA
DFR-2R	TCAATCCTACATCAAACCCGTAT
ANS-1F	CACATACTTCCTCTCAACTCACAACA
ANS-1R	AACGACGAACTGCACTCTTATTT
UF3GT-5F	CACACCACCACCAGCATCG
UF3GT-2R	TTTAATTCCAATCTATACTCCATC
MYB3-1F	ACCTCTCCTCCATGCAATATCA
MYB3-Q2R	TCCTCTTTGTATGAAACCGTGG
bHLH2-3F	CAAGACGAGGAAGACAGTGCA
bHLH2-3R	TATAACGTACAGAAATGTATAGCTG
WDR1-1F	TCACCAACTCCAAGTCCTCC
WDR1-1R	TGTCATCGTAGCAACTTCGC
WDR2-2F	CCATTCCCATTCCCCACAA
WDR2-1R	CATATTGCCCCGAATCCACA

Primers used in qPCR

Primer name	Primer sequence (5’-3’)
EF1a-Q1F	TGGTCAGACTCGTGAGCATG
EF1a-Q1R	TACTTCGGGGTTGTGGCATC
CHS-Q1F	TACTTCCGCATCACCAAGAGC
CHS-Q2R	CAATGAGGGAGCCATGTAGG
F3H-Q2F	CCTCCTTCTCCAAGACCAGG
F3H-Q2R	TCAGCATTCTTGAACCTCCC
DFR-Q1F	GAGGGGACAAAGTGAGCAAGTG
DFR-2R	TCAATCCTACATCAAACCCGTAT
ANS-Q1F	CCTTCAAGCAGCACATCCAGC
ANS-1R	AACGACGAACTGCACTCTTATTT
UF3GT-Q1F	GAAGATGGCGGAGGAGATGA

UF3GT-Q1R	TCCAGAGGTTCACTTAGGCG
bHLH2-Q2F	TCCTCTTCTGCTAACAACACCT
bHLH2-Q2R	ACTTCGACAATGCTGGCTCT
WDR1-Q1F	GAGCACTCCACCATCGTCTA
WDR1-Q1R	GCGCAGGTCCATCTTGTTC
WDR2-2F	CCATTCCCATTCCCCACAA
WDR2-Q4R	AGTTCATGGCGTAGATGTGC

Table S4 Numbers of gDNA sequences of *EuMYB3* in different sequence Groups for purple and yellow individuals. The "Purple" or "Yellow" groups are indicated in parenthesis. See Fig. 4 for sequences included in different Groups.

	Purple individuals										
Group	P02	P05	P13	P17	P19	P20	P22	P31	P32	P51	Sum
All	5	3	6	2	2	4	6	9	7	3	47
1.1 (Purple)	1	1	1	0	0	2	3	1	1	0	10
1.2 (Purple)	3	2	2	2	2	2	3	3	5	3	27
1.3 (Yellow)	0	0	0	0	0	0	0	0	0	0	0
2.1 (Yellow)	1	0	1	0	0	0	0	1	0	0	3
2.2 (Yellow)	0	0	0	0	0	0	0	0	0	0	0
2.3 (Yellow)	0	0	0	0	0	0	0	0	0	0	0
2.4 (Yellow)	0	0	1	0	0	0	0	1	0	0	2
3.1 (Yellow)	0	0	0	0	0	0	0	1	1	0	2
3.2 (Yellow)	0	0	1	0	0	0	0	1	0	0	2
3.3 (Yellow)	0	0	0	0	0	0	0	1	0	0	1

	Yellow individuals										
Group	Y01	Y03	Y04	Y05	Y06	Y07	Y09	Y10	Y14	Y15	Sum
All	8	6	6	10	8	9	6	9	7	7	76
1.1 (Purple)	0	0	0	0	0	0	0	0	0	0	0
1.2 (Purple)	0	0	0	0	0	0	0	0	0	0	0
1.3 (Yellow)	1	1	1	1	0	2	1	1	1	1	10
2.1 (Yellow)	0	0	1	0	2	0	0	1	0	0	4
2.2 (Yellow)	0	0	0	1	2	2	0	1	0	0	6
2.3 (Yellow)	0	0	0	0	2	0	0	0	0	0	2
2.4 (Yellow)	0	1	1	4	1	2	1	3	1	1	15
3.1 (Yellow)	4	1	1	3	0	2	1	1	2	3	18
3.2 (Yellow)	1	3	1	0	0	1	1	0	2	1	10
3.3 (Yellow)	2	0	1	1	1	0	2	2	1	1	11

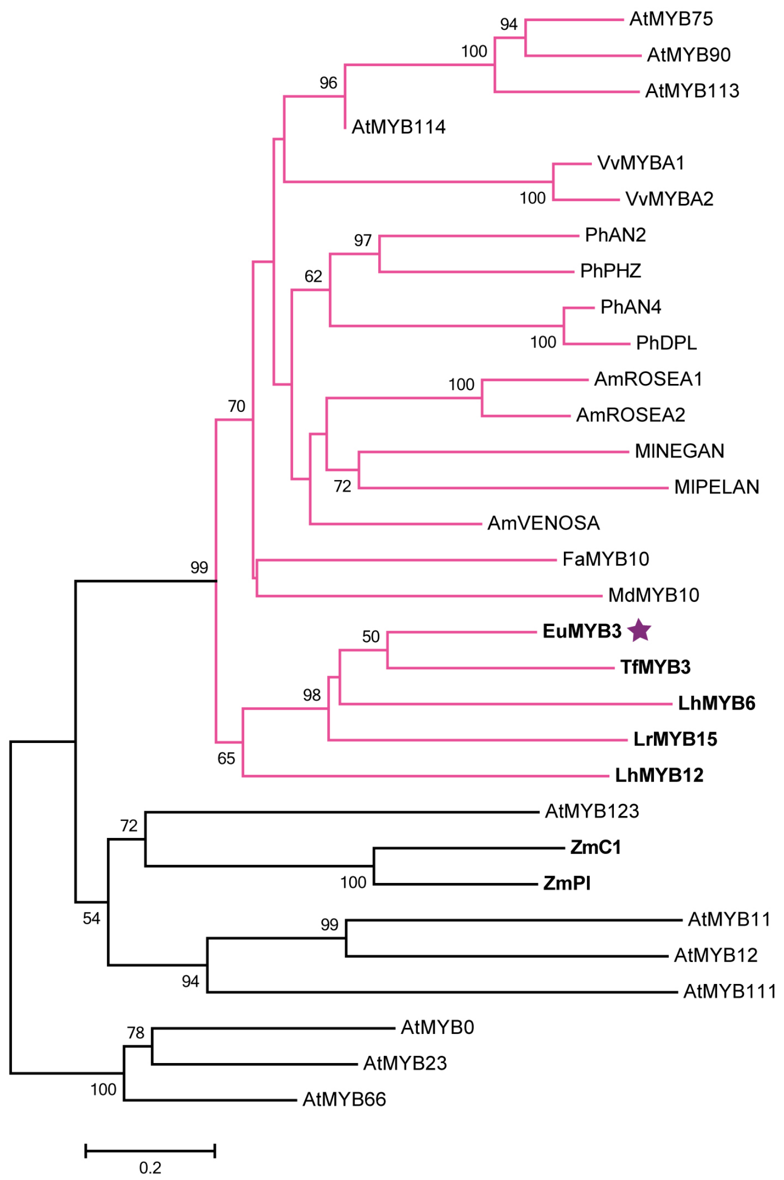

Figure S1 Neighbor-joining phylogenetic tree of R2R3-MYB proteins. The clade containing R2R3-MYBs of subgroup 6 is shown in pink. EuMYB3 from the *Erythronium umbilicatum* anther is marked with a star. The anthocyanin-regulating R2R3-MYBs from monocots are shown in bold. Bootstrap support values ≧ 50% are shown. The *Arabidopsis thaliana* sequences were retrieved from TAIR (https://www.arabidopsis.org/): subgroup 5: AtMYB123 (AT5G35550); subgroup 6: AtMYB75 (AT1G56650), AtMYB90 (AT1G66390), AtMYB113 (AT1G66370) and AtMYB114 (AT1G66380); subgroup 7: AtMYB11 (AT3G62610), AtMYB12 (AT2G47460) and AtMYB111 (AT5G49330); subgroup 15: AtMYB0 (AT3G27920), AtMYB23 (AT5G40330) and AtMYB66 (AT5G14750). Other sequences were retrieved from GenBank: *Antirrhinum majus* AmROSEA1 (ABB83826), AmROSEA2 (ABB83827), AmVENOSA (ABB83828); *Fragaria* x *ananassa* FaMYB10 (ABX79947); *Lilium* spp. LhMYB6 (BAJ05399), LhMYB12 (BAJ05398); *Lilium regale* LrMYB15 (BAU29929); *Malus domestica* MdMYB10 (ACQ45201); *Mimulus lewisii* MlPELAN (AHJ80987), MlNEGAN (AHJ80988); *Petunia* x *hybrida* PhAN2

(AAF66727), PhAN4 (ADQ00392), PhDPL (ADW94950), PhPHZ (ADW94951); *Tulipa fosteriana* TfMYB3 (AHY20034); *Vitis vinifera* VvMYBA1 (BAD18977), VvMYBA2 (BAD18978); *Zea mays* ZmC1 (P10290), ZmPl (AAA19821).

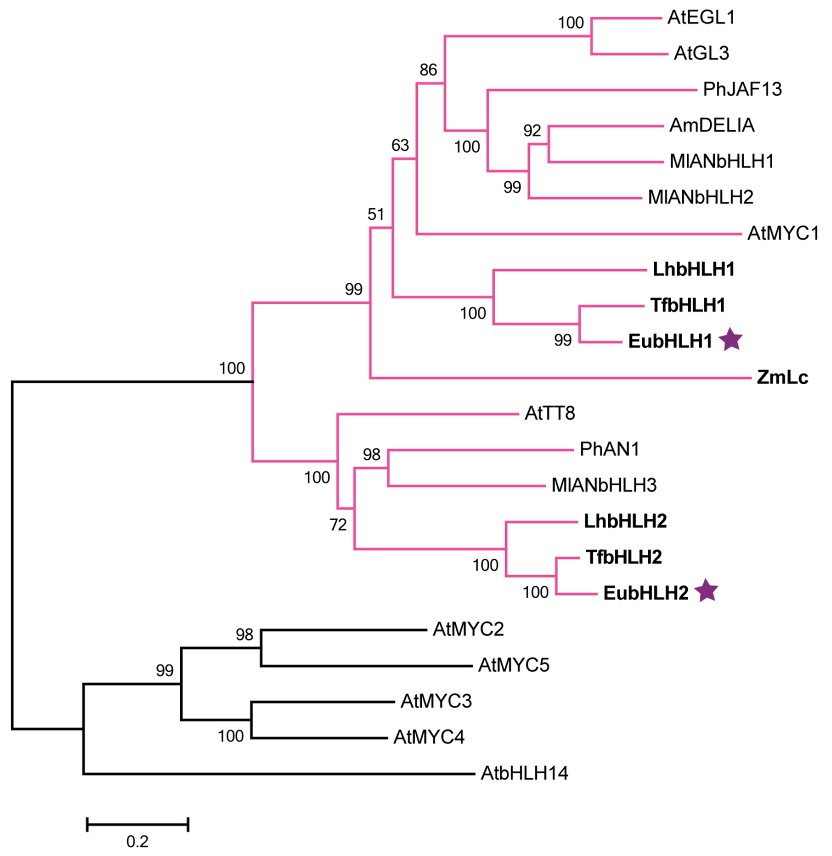

Figure S2 Neighbor-joining phylogenetic tree of bHLH proteins. The clade containing bHLHs of subgroup IIIf is shown in pink. EubHLH1 and EubHLH2 from the *Erythronium umbilicatum* anther are marked with stars. The anthocyanin-regulating bHLHs from monocots are shown in bold. Bootstrap support values ≧ 50% are shown. The *Arabidopsis thaliana* sequences were retrieved from TAIR (https://www.arabidopsis.org/): Subgroup IIIf: AtTT8 (AT4G09820), AtEGL3 (AT1G63650), AtGL3 (AT5G41315), AtMYC1 (AT4G00480); Subgroup IIIe: AtMYC2 (AT1G32640), AtMYC3 (AT5G46760), AtMYC4 (AT4G17880), AtMYC5 (AT5G46830), AtbHLH14 (AT4G00870). Other sequences were retrieved from GenBank: *Antirrhinum majus* AmDELILA (AAA32663); *Lilium* spp. LhbHLH1 (BAE20057), LhbHLH2 (BAE20058); *Mimulus lewisii* MlANbHLH1 (AHJ80985), MlANbHLH2 (AHJ80986), MlANbHLH3 (AII25878); *Petunia* x *hybrida* PhAN1 (AAG25928), PhJAF13 (AAC39455); *Tulipa fosteriana* TfbHLH1 (AGL98426), TfbHLH2 (AHY20033); *Zea mays* ZmLc (AAA33504).

```
                                                   R2 repeat
AtMYB75   ---------------MEGSSK-----GLRKGAWTTEEDSLLRQCINKYGE  30
AtMYB90   ---------------MEGSSK-----GLRKGAWTAEEDSLLRLCIDKYGE  30
AtMYB113  ---------------MGESPK-----GLRKGTWTTEEDILLRQCIDKYGE  30
AtMYB114  ---------------MEGSSK-----GLRKGAWTAEEDSLLRQCIGKYGE  30
VvMYBA1   -----------------MESL-----GVRKGAWIQEEDVLLRKCIEKYGE  28
VvMYBA2   -----------------MKSL-----GVRKGAWTQEEDVLLRKCIEKYGE  28
PhAN2     ------------MSTSNASTS-----GVRKGAWTEEEDLLLRECIDKYGE  33
PhAN4     -----------MKTSVFTSSG-----VLRKGSWTEEEDILLRKCIEKYGE  34
PhDPL     -----------MNTSVFTSSG-----VLRKGAWAEEEDILLRKCIEKYGE  34
PhPHZ     ---------MNTSSTIPKSSG-----LVRKGAWTEEEDVLLRKCIEKFGE  36
AmROSEA1  ---------------MEKNCR-----GVRKGTWTKEEDTLLRQCIEEYGE  30
AmROSEA2  ---------------MQKNPR-----GVRKGTWTKEEDILLMECIDKYGE  30
AmVENOSA  ---------------MGNNPL-----GVRKGTWTKEEDILLKQCIEKYGE  30
MlNEGAN   ---------------MENTPV-----GVRKGAWSPEEDVLLRKCIENYGE  30
MlPELAN   ---------------MEKKKVLGL--GVRKGSWTKEEDSLLRKCVETYGA  33
FaMYB10   -----------------MEGF-----GVRKGAWTKEEDELLKQFIEIHGE  28
MdMYB10   -------------MEGYNENL-----SVRKGAWTREEDNLLRQCVEIHGE  32
EuMYB3    -MSVLTTTASSTSSQPPPSLV-----ILCKGAWTTTEDDLLRRCIEKHGA  44
TfMYB3    -MSLLTTISSSSSSRLPPSSV-----LLRRGAWTQAEDDLLRRCLEKHGS  44
LhMYB6    MSPFRVSATSSSFSQMSPSPVLR---LVRKGAWTQVEDDLLKRCIERHGV  47
LhMYB12   -MFQTFIAPANTGTTSPTSAGSGGSPGTRKGQWSKEEDNLLRKCINQYNP  49
LrMYB15   -MRKMPRTMSGKTSDSPTKSQLRTSVSVRKGAWTQAEDELLRSCIEKHGT  49

                                                    R3 repeat
AtMYB75   GKWHQVPVRAGLNRCRKSCRLRWLNYLKPSIKRGKLSSDEVDLLLRLHRL  80
AtMYB90   GKWHQVPLRAGLNRCRKSCRLRWLNYLKPSIKRGRLSNDEVDLLLRLHKL  80
AtMYB113  GKWHRVPLRTGLNRCRKSCRLRWLNYLKPSIKRGKLCSDEVDLVLRLHKL  80
AtMYB114  GKWHQVPLRAGLNRCRKSCRLRWLNYLKPSIKRGKFSSDEVDLLLRLHKL  80
VvMYBA1   GKWHLVPLRAGLNRCRKSCRLRWLNYLKPDIKRGEFALDEVDLMIRLHNL  78
VvMYBA2   GKWHLVPLRAGLNRCLKSCRLRWLNYLKPDIKRGEFALDEVDLMIRLHNL  78
PhAN2     GKWHLVPVRAGLNRCRKSCRLRWLNYLRPHIKRGDFSLDEVDLILRLHKL  83
PhAN4     GKWHQVPVRAGLNRCRKSCRLRWMNYLRPHIKRGDFSPDEVDLILRLHKL  84
PhDPL     GKWHQVPVRAGLNRCRKSCRLRWLNYLRPHIKRGDFCPEEVDLIQRLHKL  84
PhPHZ     GKWHQVPVRAGLNRCRKSCRLRWLNYLRPHIKRGDFSEDEVDLILRLHKL  86
AmROSEA1  GKWHQVPHRAGLNRCRKSCRLRWLNYLRPNIKRGRFSRDEVDLIVRLHKL  80
AmROSEA2  GKWHQVPLKAGLNRCRKSCRLRWLNYLRPNIKRGCFSKDEVDLIVRLHKL  80
AmVENOSA  GKWHQVPIRAGLNRCRKSCRMRWLNYLSPNIKRGSFTRDEVDLIVRLHKL  80
MlNEGAN   GKWHLVPLRAGLNRCRKSCRLRWMNYLKPNIKRGQFNNDEVDLIIRLHKL  80
MlPELAN   GKWHLIPLRAGLNRCRKSCRLRWLNYLRPNLKRGIFDKDEIDLIVRLHKL  83
FaMYB10   GKWHHVPLKSGLNRCRKSCRLRWLNYLKPNIKRGEFAEDEVDLIIRLHKL  78
MdMYB10   GKWNQVSYKAGLNRCRKSCRQRWLNYLKPNIKRGDFKEDEVDLIIRLHRL  82
EuMYB3    VRWNRVPQLAGLNRCRKSCRLRWLNYLDPRIKRGRFEEDEDDLIFRLHKL  94
TfMYB3    LRWCHVARMAGLNRCRKSCRLRWLNYLDPRLKRGIFEEDEKDLIVRLHKL  94
LhMYB6    VRWSRVPQLAGLNRCRKSCRLRWLNYLDPRIRRGQFEEDEDDLIIRLHKL  97
LhMYB12   VKWSHVPKLAGLNRCRKSCRLRWVNYLDPSINRGSFSEDEEDLIIRLHKL  99
```

Figure S3 (to be continued on the next page)

```
                         R3 repeat
AtMYB75   LGNRWSLIAGRLPGRTANDVKNYWNTHLSKKHE-PCCKIKMKKRDITPIP 129
AtMYB90   LGNRWSLIAGRLPGRTANDVKNYWNTHLSKKHESSCCKSKMKKKNIISPP 130
AtMYB113  LGNRWSLIAGRLPGRTANDVKNYWNTHLSKKHDERCCKTKMINKNITSHP 130
AtMYB114  LGNRWSLIAGRLPGRTANDVKNYWNTHLSKKHEPCCK------------- 117
VvMYBA1   LGNRWSLIAGRLPGRTANDVKNYWHSHHFKKEV--------QFQEEGRDK 120
VvMYBA2   LGNRWSLIAGRLPGRTANDVKNYWHGHHLKKKV--------QFQEEGRKK 120
PhAN2     LGNRWSLIAGRLPGRTANDVKNYWNTHLRKKLIAPH-----DQKQESKNK 128
PhAN4     LGNRWSLIAGRLPGRTANDVKNYWNTNLLRRSKFAP-----PQQHDRKCP 129
PhDPL     LGNRWSLIAGRLPGRTANDVKNYWNTHLLRRSNFAPP----PQQHERKCT 130
PhPHZ     LGNRWSLIAGRLPGRTANDVKNYWNTHLQRKLIAP------PRQEIRKCR 130
AmROSEA1  LGNKWSLIAGRIPGRTANDVKNFWNTHVGKNLGEDG-----ERCRKNVMN 125
AmROSEA2  LGNKWSLIAGRIPGRTANDVKNFWNTHVGKNLGVDG-----ERRKKNVMN 125
AmVENOSA  LGNRWSLIAGRLPGRTGNDVKNFWNTHFEKKSG--------ERENTENIN 122
MlNEGAN   LGNRWSLIAGRLPGRTANDVKNVWNSQIEKKLLAGGGEIT-PRAAAAKVQ 129
MlPELAN   LGNRWSCIAGRIPGRTANDVKNFWNTHFKRKKKPSSSAAATTESSRRSVV 133
FaMYB10   LGNRWSLIAGRLPGRTANDVKNYWNTYQRKKDQKTAS----YAKKLKVKP 124
MdMYB10   LGNRWSLIARRLPGRTANAVKNYWNTRLRID----------SRMKTVKNK 122
EuMYB3    LGNRWSLIAGRLPGRTANDVKNYWNSHLSKKLEAG------DKKYRGRRR 138
TfMYB3    LGNRWSLIAGRVPGRTANDVKNHWNSRLNKKLVTE------ARKYGERRE 138
LhMYB6    LGNRWSLIAGRLPGRTANDVKNYWNSHLSKKLIP-------QEKKVRACP 140
LhMYB12   LGNRWSLIAGRLPGRTANDIKNYWNSHLSKRKVN---------------- 133
LrMYB15   LGNRWSLIAGRLPGRTANDVKNHWNSRLSKKLISG------IKNDGSRGR 143

                         motif 6
AtMYB75   T--TPALKNNVYKPRPRSFTVNNDCNHLNAPPKVDV------------NP 165
AtMYB90   T--TPVQKIGVFKPRPRSFSVNNGCSHLNGLPEVDL------------IP 166
AtMYB113  T--SSAQKIDVLKPRPRSFSDKNSCNDVNILPKVDV------------VP 166
AtMYB114  ---TKIKRINIITPP---------------------------------NT 131
VvMYBA1   P--QTHSKTKAIKPHPHKFS--------KALPRFEL------------KT 148
VvMYBA2   P--QTHSKTKAIKPHPHKFS--------KALPRFEL------------KT 148
PhAN2     A--VKITENNIIKPRPRTFS-RPAMNNFPCWNGKSC------------NK 163
PhAN4     KAIKTMAKNAIIRPQPRNLS---------KLAKNNV------------ST 158
PhDPL     KEIRTMAKNAIIRPQPRNLS---------KLAKNNVSNH---------ST 162
PhPHZ     A--LKITENNIVRPRPRTFS---NNAQNISWCSNKSI-----------TT 164
AmROSEA1  T--KTIKLTNIVRPRARTFT-----GLHVTWPREVGKT----------DE 158
AmROSEA2  T--KNSKETNIIRPRARTFN-----GLHVTWPREHGKND---------AF 159
AmVENOSA  P--KLINSSNIIKPQPRTFL------------KLRP------------KE 146
MlNEGAN   ---KIITSTNIVRPRPRAFS--------------NL------------SP 150
MlPELAN   V--KTITERNIIRPQPTTFSNRCRSDIQQLMTKTNETNDDDENPKINKNP 181
FaMYB10   R--ENTIAYTIVRPRPRTFI-----------KRFNF------------TE 149
MdMYB10   S--QEMRKTNVIRPQPQKFN-----------------------------R 141
EuMYB3    V-------ATPIRPQPRRWSKE--------------------------EK 155
TfMYB3    V-------APPITPQHQCFS------------------------------ 151
LhMYB6    C------IAAPTRPQPRKCSIKTKTSVDDQQVNMSELIPQKKKVRACRII 184
LhMYB12   ---VEQRTLKPIRPQPVTLP----------------------------RN 152
LrMYB15   V-------AAPIRPQPRTIP---------------VRMQ---------KT 162
```

Figure S3 (to be continued on the next page)

```
AtMYB75   PCLGLNINNVCDNSIIYNKDKKKDQLVN-NLIDGDN---MWLEK-FLEES 210
AtMYB90   SCLGLKKNNVCENSITCNKDDEKDDFVN-NLMNGDN---MWLEN-LLGEN 211
AtMYB113  LHLGLNNNYVCESSITCNKDEQKDKLININLLDGDN---MWWES-LL--- 209
AtMYB114  PAQK---------------------------------------------- 135
VvMYBA1   TAVDTFDTQVS----TSRKPSSTSPQPNDDII--------WWES-LLAEH 185
VvMYBA2   TAVDTFDTQVS----TSSKPSSTSPQPNDDII--------WWES-LLAEH 185
PhAN2     NTIDKNEGDT-----EIIKFSDEKQKPEESIDDGLQ----WWAN-LLANN 203
PhAN4     IHKDEHSKQEI----IIEKPTTAEVVSRDENVE-------WWTNLLLDNC 197
PhDPL     KHKDEYSKQKM----FIEKPTTAEVVSRDNNVE-------WWTNLLLDNC 201
PhPHZ     STIDKDGSNN-----ECIRINDKKPMAEESRHDGVQ----WWTS-LLANC 204
AmROSEA1  FSNVRLTTDEIPDCEKQTQFYNDVASPQDEVEDCIQ----WWSK-LLETT 203
AmROSEA2  SNVRITSTTENLDYEKQKPFHNNVASTPEEVDESIR----WWSNLLETTE 205
AmVENOSA  TKKQKNIRNVC-----TANDDKQQPLSTSGQLEEVNERIRWWSE-LLDFA 190
MlNEGAN   PTTNENPTKNPSST-SSLALAASSSRETDEIVR-------WWRN-LLETT 191
MlPELAN   SSSSSSSSDHSPLLRECGPVNDDRNRINGDDDDHQNDP--KKKDPLIQSS 229
FaMYB10   RYANIEHNHSEVSYTSSLPTEPPQTLQLENVTD-------WWKDFSEDST 192
MdMYB10   SSYYLSSKEPILDHIQSAEDLSTPPQTSSSTKNGND----WWET-LLEGE 186
EuMYB3    PIIDIEQQQ------QDLTLSGASAQQEDDAL--------WVESLIRDDE 191
TfMYB3    ----RKQQSSPERLQEDLNVTGVSAYQQDNT---------WVDRLLLYNE 188
LhMYB6    AAPTRPQPRKCSIETKTSVDEQQVNMSESRPSADTANCAVWQDD-LGNVK 233
LhMYB12   WSWLRMKKQ------GEAEPKMETKVPDEEEHDQ------WL---MINDS 187
LrMYB15   GHVERKHGEIQP---VSVVEEDHHTSRMENIIDDDE----NYNTKKTERQ 205


AtMYB75   QEVD---------------ILVPE--ATTTEKG---------------DT 228
AtMYB90   QEAD---------------AIVPE--ATTAEHG---------------AT 229
AtMYB113  -EAD---------------VLGPE--ATETAKG---------------VT 226
AtMYB114  -------------------------------------------------- 135
VvMYBA1   AQMDQETDFSASGEMLIASLRTEE--TATQKKGP--MDGMIEQIQGGEGD 231
VvMYBA2   AQMDQETDFSASGEMLIASLWTEE--TATQKKG---THSKTKAIKPHPHK 230
PhAN2     IEIE-----ELVSCNS-PTLLHEE--TAPSVNA---ESSLTQGGGSGLSD 242
PhAN4     NGFEKAATESTSAFKNIESLLNEELLSPSINGGT--YYPMQETRDMGWSD 245
PhDPL     NGFEKAAPESSSTFKNIESLLNEELLSASINGGT--NYPIQETGDMGWSD 249
PhPHZ     NEND-ETAVENMSYDKLPSLLHEEI-SPTINGG---ISNCMQEGQTGWDD 249
AmROSEA1  EDGE-----------------LGNLFEEAQQIGN---------------- 220
AmROSEA2  DELE-------------------NLFEDVQQTGK---------------- 220
AmVENOSA  DYVD---------------------------------------------- 194
MlNEGAN   TSTEDGILVAGEEERQTGKLCRENAADLDEEDGGAAVQEGEAEDEDGLAD 241
MlPELAN   SSQD------QADEEDEYVRWWRDLLEMTEKDHGATPLLFSDNPIIDDNN 273
FaMYB10   ESID---------------------------------RTMCSGLGLEDHD 209
MdMYB10   DTFERAAYPSIELEEELFTSFWFDDRLSPRS-----CANFPEGQSRSEFS 231
EuMYB3    NYKN------------------------------------ENMNGRGEDN 205
TfMYB3    EYNK-------------EKTEWQHLSDFSLEDVEGFKEGMMLEGNLGLDT 225
LhMYB6    EMIE----------------QLTEATIPSENTEGFAHEGLMQDGVSLWDN 267
LhMYB12   KHGH----------ENYYTMNDQMDSNQQADFGFECIYGVGEEETTVDAI 227
LrMYB15   READ---------------------FSFFDNEGFREDEWLMQDGISAWQN 234
```

Figure S3 (to be continued on the next page)

```
 AtMYB75  LA-----FDV----DQLW--SLFDGETVKFD------  248
 AtMYB90  LA-----FDV----EQLW--SLFDGETVELD------  249
AtMYB113  LP-----LDF----EQIW--ARFDEETLELN------  246
AtMYB114  --------------VDIF-------------------  139
 VvMYBA1  FP-----FD-----VGFW--DTPNTQVNHLI------  250
 VvMYBA2  FSKALPRFELKTTAVDTF--DTQVSTSSKLIHVTTTE  265
   PhAN2  FS-----VDI----DDIW--DLVS-------------  255
   PhAN4  LS-----ID-----ADLW--ELL--------------  256
   PhDPL  FC-----ID-----SDPW--ELLLQ------------  262
   PhPHZ  FS-----VDI----DHLW--NLLN-------------  262
AmROSEA1  -------------------------------------  220
AmROSEA2  --------------MSEW-------------------  224
AmVENOSA  -------------------------------------  194
 MlNEGAN  LL-----LD-----VDIWELLSFDDERDDSWGLLGPN  268
 MlPELAN  YA-IDDGLSSGLCLDDVW--DLLSSHDH---------  298
 FaMYB10  FF------------TNFWVEDMLLSASNDLVNISYV-  233
 MdMYB10  FS------------TDLW--NHSKEE-----------  243
  EuMYB3  FN-----LEGMEGFTEFW--NNLISDMPL--------  227
  TfMYB3  FL-----SD-----MQLWS------------------  234
  LhMYB6  FI-----FDI----------QLSS-------------  276
 LhMYB12  LQ-----WDGLLSDIKLWSDSEVV-------------  246
 LrMYB15  LL------------SDLL--TGG--------------  243
```

Figure S3 Alignment of anthocyanin-regulating R2R3-MYB proteins (subgroup 6)[1]. If > 70% of amino acids at each column are identical, this common is highlighted in a black background, and its similar amino acids are highlighted in a gray background. The domains of R2 and R3 repeats and the motif of subgroup 6 are indicated as bars above the alignment. The motif 6 was defined as "KPRPR[S/T]F" based only on the *Arabidopsis* sequences[1]. However, our data show "[K/R]P[R/Q]PR" is a more generalized version of this motif. The bHLH interaction domain "[D/E]Lx_2[R/K]$x_3Lx_6Lx_3$R"[2] located in the R3 repeat is indicated in yellow letters.

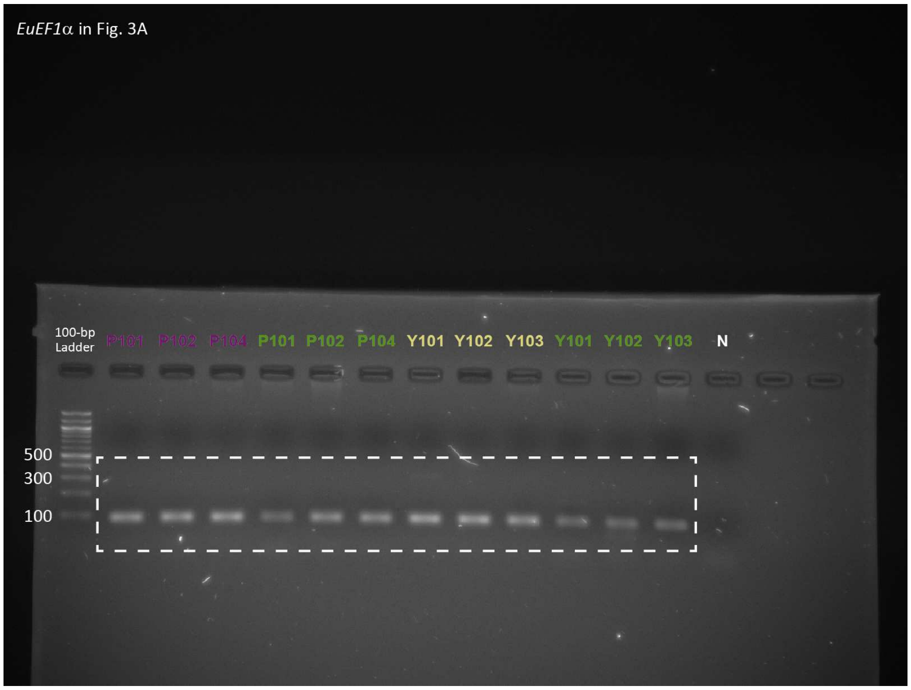

Figure S4 The original gel photograph that was cropped to create the panel Fig. 3A "*EuEF1*α". Dotted rectangle indicates the area shown in Fig. 3A. The100-bp DNA ladder, Plant IDs (referring to Fig.3 for sample information), and a negative control (N) are showed at the top of each lane. The 100-bp, 300-bp and 500-bp fragments of the ladder are labeled.

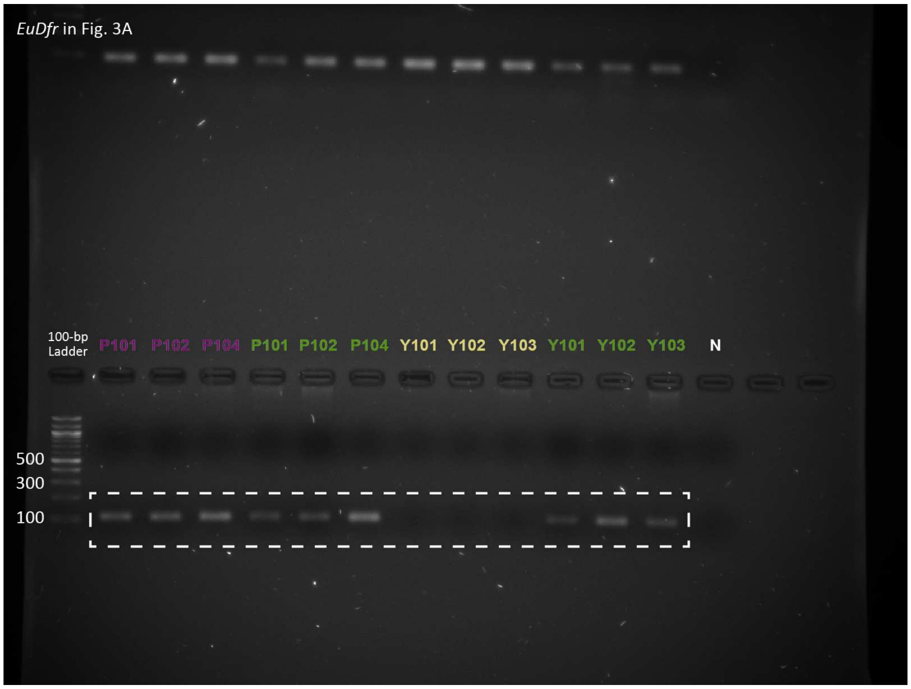

Figure S5 The original gel photograph that was cropped to create the panel Fig. 3A "*EuDfr*". Dotted rectangle indicates the area shown in Fig. 3A. The100-bp DNA ladder, Plant IDs (referring to Fig.3 for sample information), and a negative control (N) are showed at the top of each lane. The 100-bp, 300-bp and 500-bp fragments of the ladder are labeled.

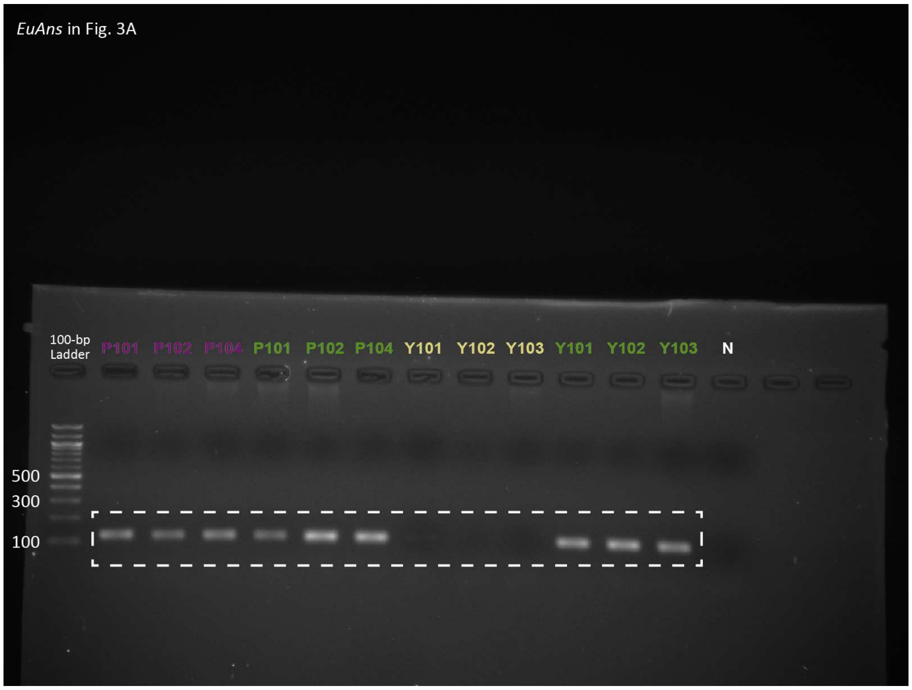

Figure S6 The original gel photograph that was cropped to create the panel Fig. 3A "*EuAns*". Dotted rectangle indicates the area shown in Fig. 3A. The100-bp DNA ladder, Plant IDs (referring to Fig.3 for sample information), and a negative control (N) are showed at the top of each lane. The 100-bp, 300-bp and 500-bp fragments of the ladder are labeled.

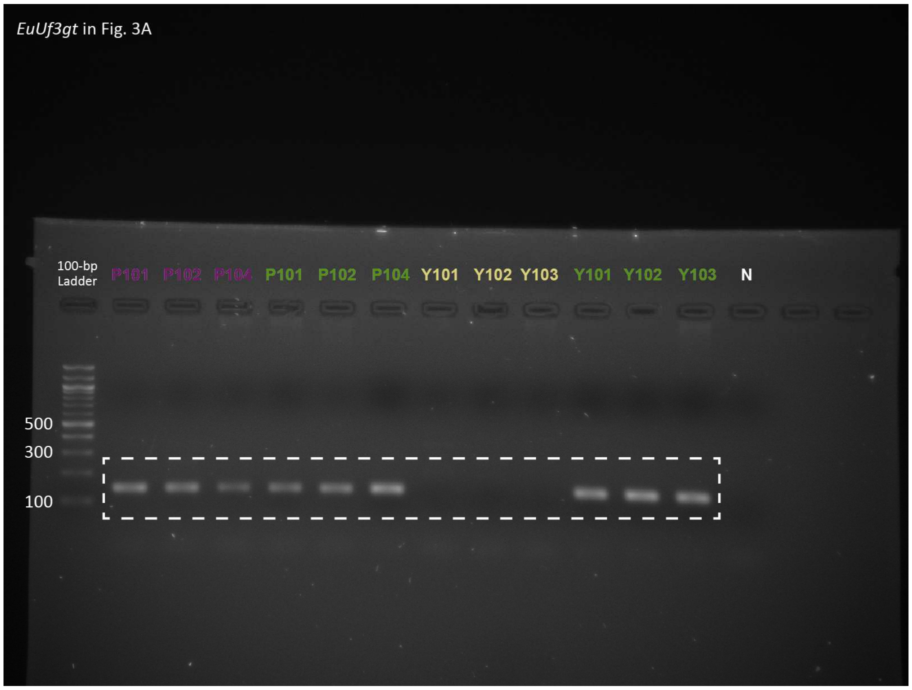

Figure S7 The original gel photograph that was cropped to create the panel Fig. 3A "*EuUf3gt*". Dotted rectangle indicates the area shown in Fig. 3A. The100-bp DNA ladder, Plant IDs (referring to Fig.3 for sample information), and a negative control (N) are showed at the top of each lane. The 100-bp, 300-bp and 500-bp fragments of the ladder are labeled.

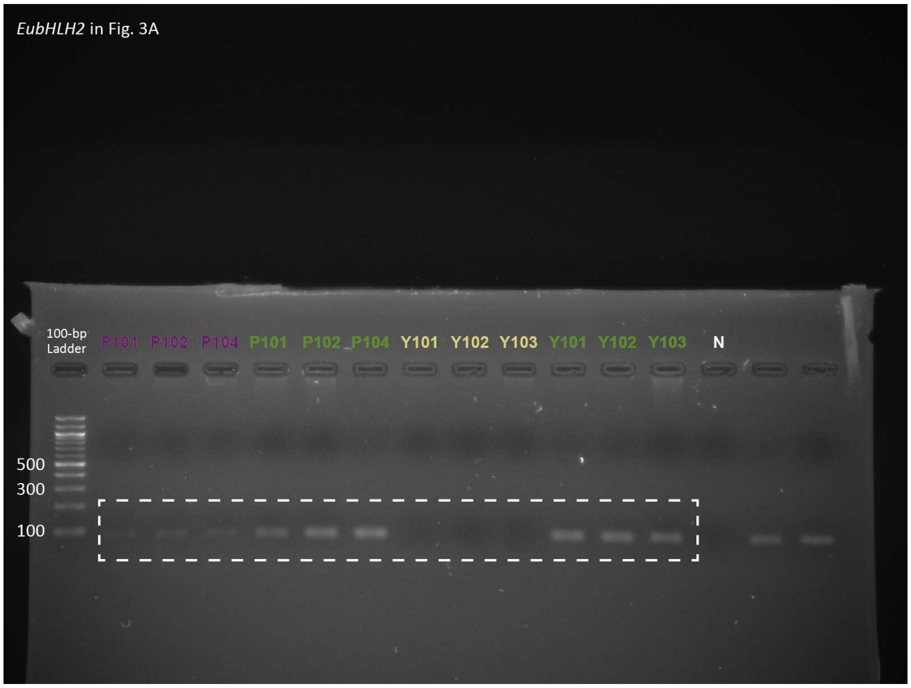

Figure S8 The original gel photograph that was cropped to create the panel Fig. 3A "*EubHLH2*". Dotted rectangle indicates the area shown in Fig. 3A. The100-bp DNA ladder, Plant IDs (referring to Fig.3 for sample information), and a negative control (N) are showed at the top of each lane. The 100-bp, 300-bp and 500-bp fragments of the ladder are labeled. The last two lanes are samples for the other experiment.

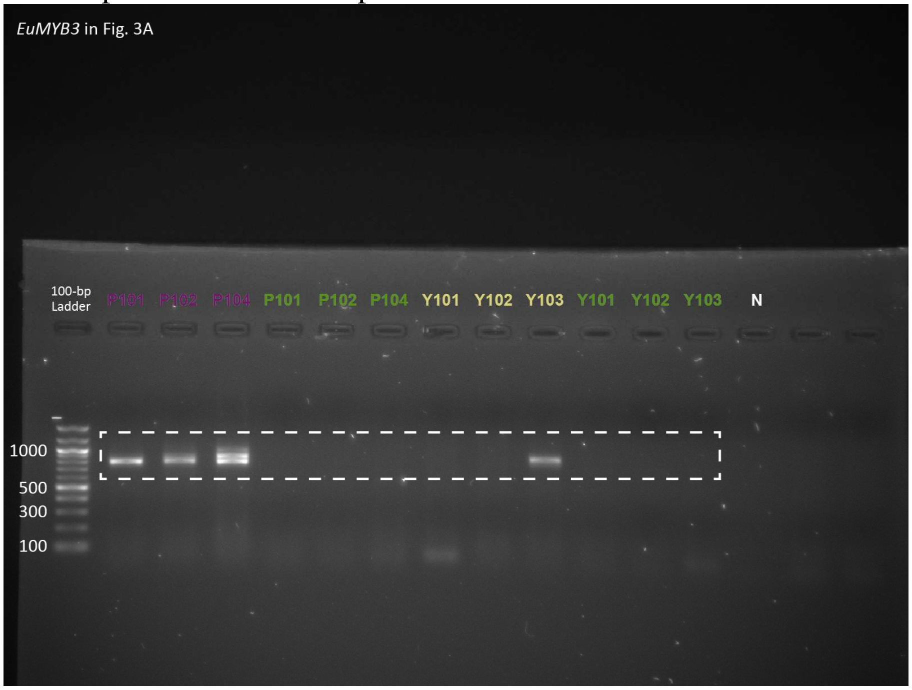

Figure S9 The original gel photograph that was cropped to create the panel Fig. 3A "*EuMYB3*". Dotted rectangle indicates the area shown in Fig. 3A. The100-bp DNA ladder, Plant IDs (referring to Fig.3 for sample information), and a negative control (N) are showed at the top of each lane. The 100-bp, 300-bp, 500-bp and 1000-bp fragments of the ladder are labeled.

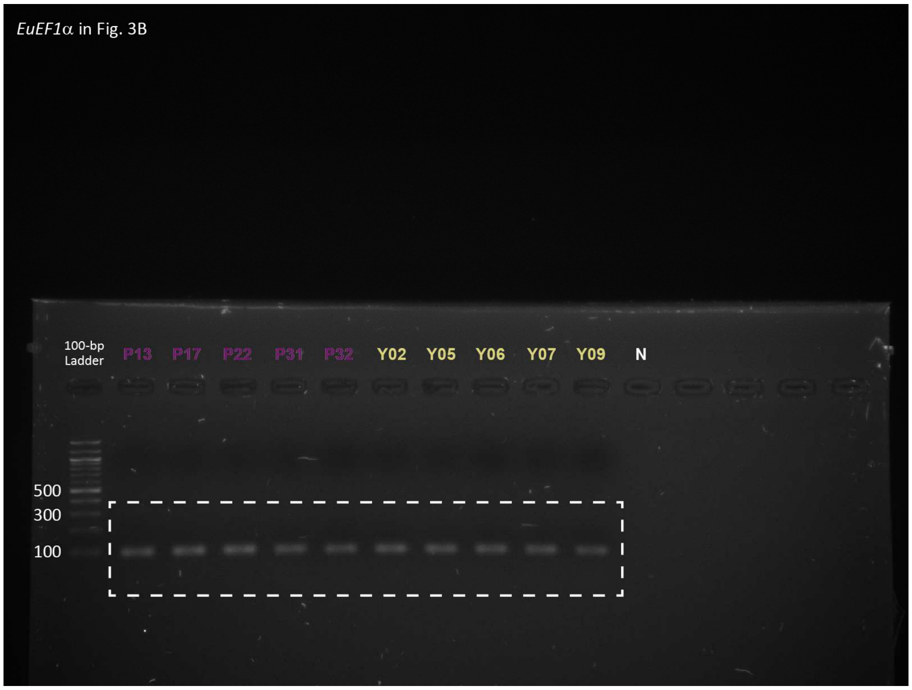

Figure S10 The original gel photograph that was cropped to create the panel Fig. 3B "*EuEF1*α". Dotted rectangle indicates the area shown in Fig. 3B. The100-bp DNA ladder, Plant IDs (referring to Fig.3 for sample information), and a negative control (N) are showed at the top of each lane. The 100-bp, 300-bp and 500-bp fragments of the ladder are labeled.

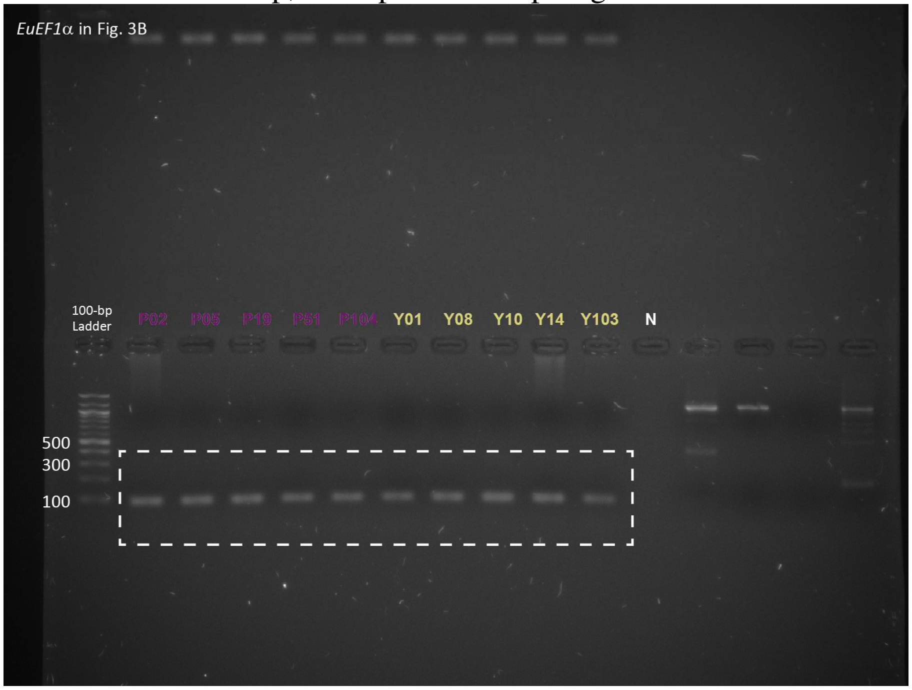

Figure S11 The original gel photograph that was cropped to create the panel Fig. 3B "*EuEF1*α". Dotted rectangle indicates the area shown in Fig. 3B. The100-bp DNA ladder, Plant IDs (referring to Fig.3 for sample information), and a negative control (N) are showed at the top of each lane. The 100-bp, 300-bp and 500-bp fragments of the ladder are labeled. The last four lanes are samples for the other experiment.

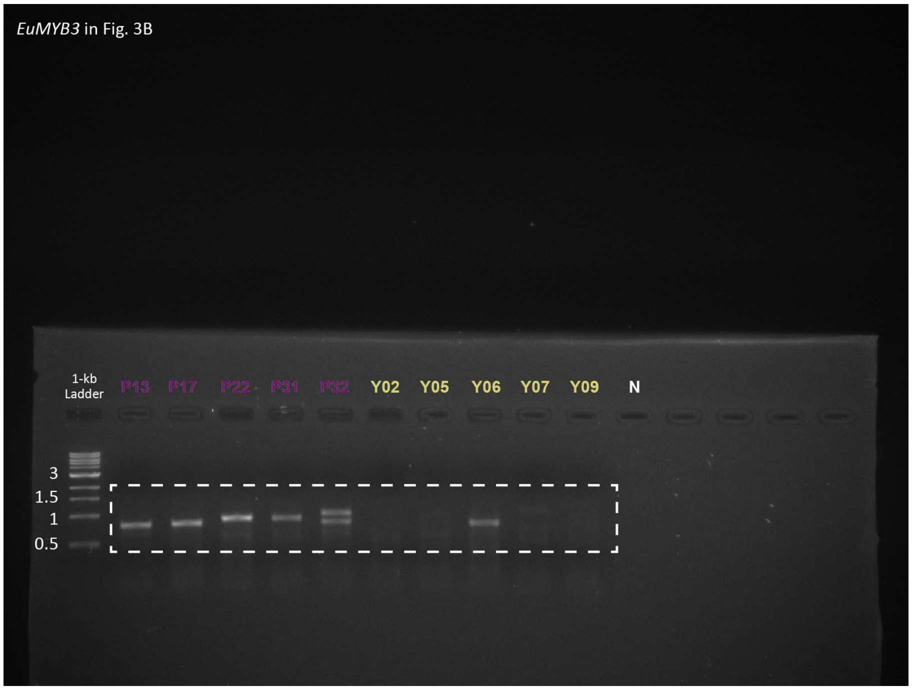

Figure S12 The original gel photograph that was cropped to create the panel Fig. 3B "*EuMYB3*". Dotted rectangle indicates the area shown in Fig. 3B. The 1-kb DNA ladder, Plant IDs (referring to Fig.3 for sample information), and a negative control (N) are showed at the top of each lane. The 0.5-kb, 1-kb, 1.5-kb and 3-kb fragments of the ladder are labeled.

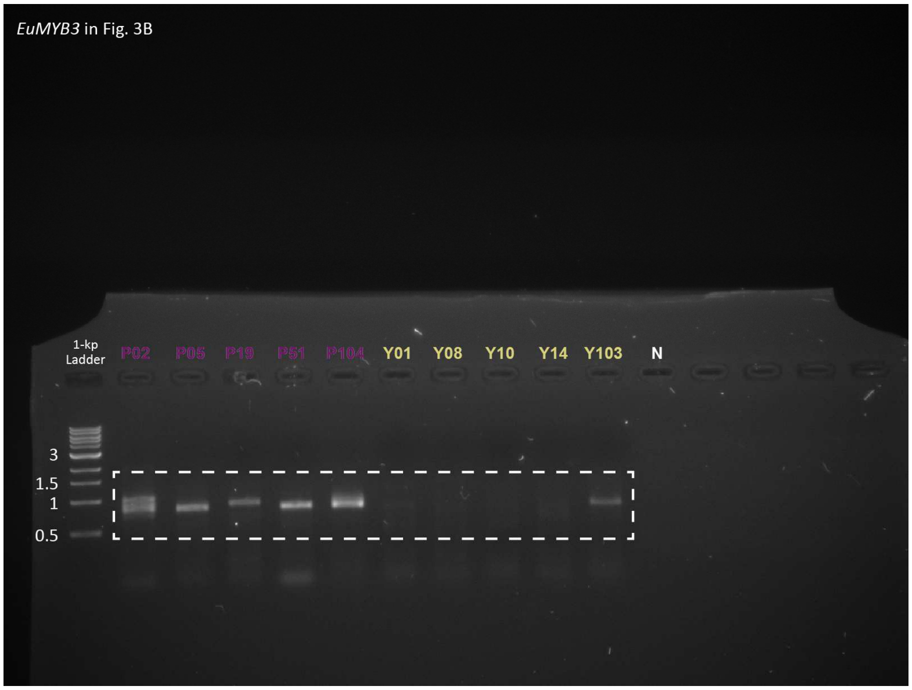

Figure S13 The original gel photograph that was cropped to create the panel Fig. 3B "*EuMYB3*". Dotted rectangle indicates the area shown in Fig. 3B. The 1-kb DNA ladder, Plant IDs (referring to Fig.3 for sample information), and a negative control (N) are showed at the top of each lane. The 0.5-kb, 1-kb, 1.5-kb and 3-kb fragments of the ladder are labeled.

Methods S1 Likelihood estimation of joint *cis*-regulatory mutations leading to triple downregulation

Our results indicate that three enzyme-coding genes (*EuDfr*, *EuAns* and *EuUf3gt*) are downregulated in the yellow anthers of *Erythronium umbilicatum*. One explanation of such downregulation is independent *cis*-regulatory mutations occurring in all three genes. We thus calculated the maximum probability that a yellow-anthered individual was homozygous for the downregulated "yellow" allele at each of the three loci with the phenotype frequency data from *E. umbilicatum* (Supplementary Table S1).

We assume that there is no linkage disequilibrium between *EuDfr*, *EuAns*, and *EuUf3gt*, which allows the expected frequencies of three-locus genotypes to be computed as the product of the individual single-locus genotypes. This assumption seems reasonable for long-standing natural populations. Let *D* and *d*, *A* and *a*, and *U* and *u* be the alternative alleles at *EuDfr*, *EuAns*, and *EuUf3gt*, respectively, where lower-case alleles correspond to the downregulated "yellow" alleles (i.e., individuals that are *dd*, *aa*, or *uu* have yellow anthers). Let *q*, *s*, and *v* be the frequencies of *d*, *a*, and *u*, respectively. Then the expected frequencies of the different yellow-anthered genotypes are:

$Freq(ddA\_U\_) = q^2 (1 - s^2) (1 - v^2)$

$Freq(ddaaU\_) = q^2 s^2 (1 - v^2)$

$Freq(ddA\_uu) = q^2 (1 - s^2) v^2$

$Freq(ddaauu) = q^2 s^2 v^2$

$Freq(D\_aaU\_) = (1 - q^2) s^2 (1 - v^2)$

$Freq(D\_aauu) = (1 - q^2) s^2 v^2$

$Freq(D\_A\_uu) = (1 - q^2) (1 - s^2) v^2$.

The sum of these frequencies is the expected frequency of yellow-anthered individuals, $Freq(Y)$, which equals to $q^2 (1 - s^2) (1 - v^2) + s^2 + v^2 - s^2v^2$. The probability that a yellow-anthered individual is homozygous for the "yellow" alleles at all three loci (i.e., the genotype *ddaauu*) is then $Prob(ddaauu) = q^2 s^2 v^2 / Freq(Y)$.

Although this probability depends on the allele frequencies, we obtained a conservative estimate by finding its maximum, $P_{\max}$, using the FindMaximum function in MATHEMATICA 12 (Wolfram Research, Champaign, IL, USA). $P_{\max}$ is subject to the constraint $q^2 (1 - s^2) (1 - v^2) + s^2 + v^2 - s^2v^2 = Freq(Y)$, where $Freq(Y)$ equals to 0.183 as the frequency of the yellow-anthered individuals in the Oosting Natural Area (Supplementary Table S1). The maximum of

Prob(*ddaauu*) was estimated to be 0.00151 when the frequencies of *s* and *v* were 0.2553. Thus, the probability that all 10 sampled individuals are *ddaauu* is less than or equals to $(0.00151)^{10} = 6.21 \times 10^{-29}$. Because of this extremely small value, we reject the hypothesis that this triple downregulation is caused by independent *cis*-regulatory mutations in *EuDfr*, *EuAns*, and *EuUf3gt*.

Methods S2 Phylogenetic analyses of R2R3-MYB and bHLH proteins

To evaluate whether *EuMYB3*, *EubHLH1* and *EubHLH2* are homologs to the known anthocyanin regulators, we constructed a phylogenetic tree of EuMYB3 and the related R2R3-MYB proteins, and also a phylogenetic tree of EubHLHs and the related bHLH proteins. Because multiple copies of *EuMYB3* were detected, we chose the most frequent copy of *EuMYB3* (GenBank accession number: OK648453) for the analyses. The coding sequences of *EuMYB3*, *EubHLH1* (transcriptome contig ID: EuP_21744_c0_g1_i1) and *EubHLH2* (GenBank accession number: OK648452) were translated into amino acid sequences, and then aligned with other related R2R3-MYB or bHLH protein sequences using MUSCLE[3]. The neighbor-joining phylogenetic trees were constructed using MEGA 6.06[4] with the JTT amino acid substitution model. Clade support was estimated by 1,000 bootstrap replicates.

Methods S3 Calculation of relative expression in qPCR analyses

The relative expression ratio (R) was calculated using the equation 1 in ref[5]. For this analysis, one purple cDNA sample was designated as a "control", and all other samples to be analyzed are "samples". In each qPCR run, the target gene and the reference gene were amplified from all samples including the "control". In our qPCR assays, *EF1α* (*elongation factor 1-alpha*) was used as the reference gene. The ratio was quantified with the equation

$$R = \frac{(E_{target})^{\Delta CP_{target}(control-sample)}}{(E_{ref})^{\Delta CP_{ref}(control-sample)}},$$

where E_{target} is the real-time qPCR efficiency of the target gene transcript; E_{ref} is the real-time qPCR efficiency of the *EF1α* transcript; $\Delta CP_{target}(control - sample)$ is the difference in the threshold values between the control and one sample while amplifying the target gene; $\Delta CP_{ref}(control - sample)$ is the difference in the threshold values between the control and one

sample while amplifying *EF1α*. We obtained the threshold values as the Cq values from the instrument Roche LightCycler 96. The relative expression levels were then calculated as the logarithm of the ratios, $Log_{10}(R)$.

Dataset S1 EuMYB3 amino-acid sequences with premature stop codons (indicated by *). The sequence C_P02_1 is from the purple Group 1.2 and is provided as a reference as an exemplar with no premature stop codons. All remaining sequences are from the yellow Groups. Genbank accession numbers for their nucleotide sequences are shown with the sequence names.

Group 1.2 (Purple)

>C_P02_1|OK648453
```
MSVLTTTASSTSSQPPPSLVILCKGAWTTTEDDLLRRCIEKHGAVRWNRVPQLAGLNRCRKSCRLRWLNYLDPRIKRG
RFEEDEDDLIFRLHKLLGNRWSLIAGRLPGRTANDVKNYWNSHLSKKLEAGDKKYRGRRRVATPIRPQPRRWSKEEKP
IIDIEQQQQDLTLSGASAQQEDDALWVESLIRDDENYKNENMNGRGEDNFNLEGMEGFTEFWNNLISDMPL
```

Group 2.2 (Yellow)

>G_Y05_55|OP963297
```
MSALTTTASSTSSQPPPSLVLLRKGAWTTAEDDLLRKCLEKHGAVRWNRVPQLAGLNRFRKSCRLRWLNYLDPRIKRG
RFEEDEDDLIFRLHKLLGNRWSLIAGRLPGRTANDV*NYWNSHLSKKLEAGDKKYRGRRRVATPIRRQPRRWSREEKP
SIDIEQ*QQDLTLSGASAQQEDDALSVESLISDDENYKNENMNGRGEDNLDLEDMEGFREI*NNLISDMPL*
```

>G_Y06_58|OP963300
```
MSALTTTASSTSSQPPPSLVLLRKGAWTTAEDDLLRKCLEKHGAVRWNRVPQLAGLNRCRKSCRLRWLNYLDPRIKRE
RFEEDEDDLIFRLHKLLGNRWSLIAGRLPGRTANDVKNHWNSHLSKKLDAGDQKFRERRRVATPIRPQPRRWSREEKP
SIDIEQQQQDLTLSGA*AQQEDDALWVESLISDDENYKNENMNGRGEDNFNLEDMEGFTEIWNNLISDMPL*
```

>G_Y06_59|OP963301
```
MSALTTTASSTSSQPPPSLVLLRKGAWTTAEDDLLRKCLEKHGAVRWNRVPQLAGLNRFRKSCRLRWLNYLDPRIKRG
RFEEDEDDLIFRLHKLLGNRWSLIAGRLPGRTANDV*NYWNSHLSKKLEAGDKKYRGRRRVATPIRRQPRRWSREEKP
SIDIEQ*QQDLTLSGASAQQEDDALSVESLISDDENYKNENMNGRGEDNLNLEDMEGFREI*NNLISDMPL*
```

>G_Y07_63|OP963305
```
MSALTTTASSTSSQPPPSLVLLRKGAWTTAEDDLLRKCLEKHGAVRWNRVPQLAGLNRFRKSCRLRWLNYLDPRIKRG
RFEEDEDDLIFRLHKLLGNRWSLIAGRLPGRTANDV*NYWNSHLSKKLEAGDKKYRGRRRVATPIRRQPRRWSREEKP
SIDIEQ*QQDLTLSGASAQQEDDALSVESLISDDENYKNENMNGRGEDNLNLEDMEGFREI*NNLISDMPL*
```

>G_Y07_64|OP963306
```
MSALTTTASSTSSQPPPSLVLLRKGAWTTTEDDLLRKCLEKHGAVRWNRVPQLAGLNRFRKSCRLRWLNYLDPRINRG
RFEEDEDDLIFRLHKLLGNRWSLIAGRLPGRTANDV*NYWNSHLSKKLEAGDKKYRGRRRVATPIRRQPRRWSREEKP
SIDIEQ*QQDLTLSGASAQQEDDALWVESLISDDENYKNENMNGRGEDNFNLEDMEGFTEIWNNLISDMPL*
```

>G_Y10_67|OP963309
```
MSALTTTASSTSSQPPPSLVLLRKGAWTTAEDDLLRKCLEKHGAVRWNRVPQLAGLNRFRKSCRLRWLNYLDPRIKRG
RFEEDEDDLIFRLHKLLGNRWSLIAGRLPGRTANDVKNYWNSHLSKKLEAGDKKYRGRRRVATPIRRQPRRWSREEKP
SIDIEQ*QQDLTLSGASAQQEDDALSVESLISDDENYKNENMNGRGEDNLNLEDMEGFREI*NNLISDMPL*
```

Group 2.3 (Yellow)

>G_Y06_61|OP963303
```
MSALTTTASSTSSQPPPSLVLLRKGAWTTTEDDLLRKCIEKHGAVRWNRVPQLAGLNRCRKSCRLRWLNYLDPRIKRG
RFEEDEDDLIFRLHKLLGKRWSLIAGRLPGRTANDV*NYWNSHLSKKLEAGDKKYRGRRRVATPIRRQPRRWSREEKP
SIDIEQ*QQDLTLSGASAQQEDDALSVESLISDDENYKNENMNGRGEDNLNLEDMEGFREI*NNLISDMPL*
```

>G_Y06_62|OP963304
```
MSALTTTASSTSSQPPPSLVLLCKGAWTTTEDDLLIKCIEKHGAVRWNRVPQLASLNRCRKSCRLRWLNYLDPRIKRG
RFEEDEDDLIFRLHKLLGKRWSLIAGRLPGRTANDV*NYWNSHLSKKLEAGDKKYRGRRRVATPIRRQPRRWSREEKP
SIDIEQ*QQDLTLSGASAQQEDDALSVESLISDDENYKNENMNGRGEDNLNLEDMEGFREI*NNLISDMPL*
```

Group 2.4 (Yellow)
>C_P13_20|OP963342
MSALTTTASSSSSQPPPSLVLLRKGAWTTTEDDLLRKCLEQHGAVRWNRVPQLAGNASLGPKLTRKCIFTSSCDILIF
PRPQ*MPEKLSTEMAQLSRPAD*AREVRRGRG*SYLQAS*ALG*QVVADSGPTSGQDSE*REELLELTLKQETRSRR*
KISRASKSCDTDQTTTSKMVERGKTEH*H*AATTGLDVEWSISSTQG*CIVGGKFDK***KL*KRKYELSR*RQLQFR
GYGRIYRNLE*FDI*YATLX

>C_Y06_1|OP963376
MSALTTTASSTSSQPPPSLVLLRKGAWTTTEDDLLRKCIEKHGAVRWNRVPQLAGLNRCRKSCRLRWLNYLDPRIKRG
RFEEDEDDLIFRLHKLLGNRWSLIAGRLPGRTANDVKNYWNSHLSKKLEAGDKKISRASKSCDTDQTTTSKMVERGKT
EH*H*AATTGLDVEWSISSTRG*CIVGGKFDK***KL*KRKYELSR*RQLQFRGYGRIYRILE*FDI*YATLX

>C_Y06_2|OK648455
MSALTTTASSTSSQPPPSLVLLRKGAWTTTEDDLLRKCIEKHGAVRWNRVPQLAGNASLGLKLTRKCILTSSCDILIF
PRPQ*MPEKLSTEMAQLSRPAD*AREVRRGRG*SYLQAS*ALG*QVITMNKLEQPPLKREESLPPSLLHNFYSLM*IL
TSFFRWSLIAGRLPGRTANDVKNYWNSHLSKKLEAGDKKISRASKSCDTDQTTTSKMVERGKTEH*H*AATTGLDVEW
SISSTRG*CIVGGKFDK***KL*KRKYELSR*RQLQFRGYGRIYRILE*FDI*YATLX

>C_Y103_1| OK648456
MSALTTTASSSSSQPPPSLVLLRKGAWTTTEDDLLRKCLEQHGAVRWNRVPQLAGNASLGPKLTRKCIFTSSCDILIF
PRPQ*MPEKLSTEMAQLSRPAD*AREVRRGRG*SYLQAS*ALG*QVVADSGPTSGQDSE*REELLELTLKQETRSRR*
KISRASKSCDTDQTTTSKMVERGKTEH*H*AATTGLDVEWSISSTQG*CIVGGKFDK***KL*KRKYELSR*RQLQFR
GYGRIYRNLE*FDI*YATLX

>C_Y103_2| OP963377
MSALTTTASSSSSQPPPSLVLLRKGAWTTTEDDLLRKCLEQHGAVRWNRVPQLAGLNRCRKSCRLRWLNYLDPRIKRG
RFEEDEDDLIFRLHKLLGNRWSLIAGRLPGRTANDVKNYWNSHLSKKLEAGDKKYRGRRRVATPIRPQPRRWSREEKP
SIDIEQQQQDLTLSGA*AQHKDDALWVESLISDDENYKNENMNCRGEDNFNLEDMEGFTEIWNNLISDMPL

>G_P13_12|OP963207
MSALTTTASSSSSQPPPSLVLLRKGAWTTTEDDLLRKCLEQHGAVRWNRVPQLAGLNRCRKSCRLRWLNYLDPRIKRG
RFEEDEDDLIFRLHKLLGNRWSLIAGRLPGRTANDVKNYWNSHLSKKLEAGDKKYRGRRRVATPIRPQPRRWSREEKP
SIDIEQQQQDLTLSGA*AQHKDDALWVESLISDDENYKNENMNCRGEDNFNLEDMEGFTEIWNNLISDMPL*

>G_P31_29|OP963224
MSALTTTASSTSSQPPPSLVLLGKGAWTTTEDDLLRKCLEKRGAVRWNRVPQLAGLNRCRKSCRLRWLNYLDPRIKQG
RFEEDEDDVVADSGQTSGQDSE*REELLELTLKQETRSRR*KISRASKSCDTDQTTTSKMVERGKTEH*H*AATTRLD
VEWSISSTRG*CIVGGKFDK***KL*KRKYELSR*RQLQFRGYGRIYRILE*FDI*YATLX

>G_Y03_2|OP963244
MSALTTTASSTSSQPPRSLVLLGKGAWTTTEDDLLRKCLEKHGAVRWNRVPQLAGLNRCRKSCRLRWLNYLDPRIKQG
RFEEDEDDLISSGR**RADFRAGQRMT*RTTGTHT*ARNSKPEIKNIEGVEELRHRSDHNLEDGRERKNRALTLSSNN
KT*R*VEHKLNKRMMHCGWKV**VMMKTIKTKI*TVEVKTTSI*RIWKDLQNFGII*YLICHFX

>G_Y05_3|OP963245
MSALTTTASSTSSQPPRSLVLLGKGAWTTTEDDLLRKCLEKHGAVRWNRVPQLAGLNRCRKSCRLRWLNYLDPRIKQG
RFEEDEDDLISSGR**RADFRAGQRMT*RTTGTHT*ARNSKPEIKNIEGVEELRHRSDHNLEDGRERKNRALTLSSNN
KT*R*VEHKLNKRMMHCGWKV**VMMKTIKTKI*TVEVKTTSI*RIWKDLQNFGII*YLICHFX

>G_Y09_4|OP963246
MSALTTTASSTSSQPPRSLVLLGKGAWTTTEDDLLRKCLEKHGAVRWNRVPQLAGLNRCRKSCRLRWLNYLDPRIKQG
RFEEDEDDLISSGR**RADFRAGQRMT*RTTGTHT*ARNSKPEIKNIEGVEELRHRSDHNLEDGRERKNRALTLSSNN
KT*R*VEHKLNKRMMHCGWKV**VMMKTIKTKI*TVEVKTTSI*RIWKDLQNFGII*YLICHFX

>G_Y14_5|OP963247
MSALTTTASSTSSQPPRSLVLLGKGAWTTTEDDLLRKCLEKHGAVRWNRVPQLAGLNRCRKSCRLRWLNYLDPRIKQG
RFEEDEDDLISSGR**RADFRAGQRMT*RTTGTHT*ARNSKPEIKNIEGVEELRHRSDHNLEDGRERKNRALTLSSNN
KT*R*VEHKLNKRMMHCGWKV**VMMKTIKTKI*TVEVKTTSI*RIWKDLQNFGII*YLICHFX

>G_Y15_6|OP963248
MSALTTTASSTSSQPPRSLVLLGKGAWTTTEDDLLRKCLEKHGAVRWNRVPQLAGLNRCRKSCRLRWLNYLDPRIKQG
RFEEDEDDLISSGR**RADFRAGQRMT*RTTGTHT*ARNSKPEIKNIEGVEELRHRSDHNLEDGRERKNRALTLSSNN
KT*R*VEHKLNKRMMHCGWKV**VMMKTIKTKI*TVEVKTTSI*RIWKDLQNFGII*YLICHFX

>G_Y04_7|OP963249
MSALKITVSSTSSQPPPSLVLLGKGAWTTTEDDLLRKCLEKHGAVRWNRVPQLAGLNRCRKSCRLRWLNYLDPRIKQG
RFEEDEDDLISSGR**RADFRAGQRMT*RTTGTHT*ARNSKPEIKNIEGVEELRHRSDHNLEDGRERKNRALTLSSNN
KT*R*VEHKLNKRMMHCGWKV**VMMKTIKTKI*TVEVKTTSI*RIWKDLQNFGII*YLICHFX

>G_Y05_8| OP963250
MSALTTTASSTSSQPPRSLVLLGKGAWTTTEDDLLRKCLEKHGAVRWNRVPQLAGLNRCRKSCRLRWLNYLDPRIKQG
RFEEDEDDLISSSR**RADFRAGQRMT*RTTGTHT*ARNSKPEIKNIEGVEELRHRSDHNLEDGRERKNRALTLSSNN
KT*R*VEHKLNKRMMHCGWKV**VMMKTIKTKI*TVEVKTTSI*RIWKDLQNFGII*YLICHFX

>G_Y05_54|OP963296
MSALTTTASSTSSQPPPSLVLLRKGAWTTTEDDLLRKCLEKHGAVRWNRVPQLAGPNRCRKSCRLRWLNYLDPRIKRG
RFEEDGDDLIFRLHKLLGNRWSLIAGRLPGRTANDVKNYWNSHLSKKLEAGDKKYRGRRRVATLIRPQPRRWSREEKP
SIDIEQQQQDLTLSGA*AQQEDDALWVESLISDDENYKNENMNGRGEDNFNLEDMEGFTEIWNNLISDMPL*

>G_Y06_57|OP963299
MSALTTTASSTSSQPPPSLVLLRKGAWTTTEDDLLRKCLEKHGAVRWNRVPQLAGLNKCRKSCRLRWLNYLDPRIKRG
RFEEDGDDLIFRLHKLLGNRWSLIAGRLPGRTANDVKNYWNSHLSKKLEAGDKKYRGRRRVATLIRPQPRRWSREEKP
SIDIEQQQQDLTLSGA*AQQEDDALWVESLISDDENYKNENMNGRGEDNFNLEDMEGFTEIWNNLISDMPL*

>G_Y10_65|OP963307
MSALTTTASSTSSQPPPSLVLLRKGAWTTTEDDLLRKCLEKHGAVRWNRVPQLAGLNRCRKSCRLRWLNYLDPRIKRG
RFEEDGDDLIFRLHKLLGNRWSLIAGRLPGRTANDVKNYWNSHLSKKLEAGDKKYRGRRRVATLIRPQPRRWSREEKP
SIDIEQQQQDLTLSGA*AQQEDDALWVESLISDDENYKNENMNGRGEDNFNLEDMEGFTEIWNNLISDMPL*

>G_Y10_66|OP963308
MSALTTTASSTSSQPPPSLVLLRKGAWTTTEDDLLRKCLEKHGAVRWNRVPQLAGPNRCRKSCRLRWLNYLDPRIKRG
RFEEDGDDLIFRLHKLLGNRWSLIAGRLPGRTANDVKNYWNSHLSKKLEAGDKKYRGRRRVATLIRPQPRRWSREEKP
SIDIEQQQQDLTLSGA*AQQEDDALWVESLISDDENYKNENMNCRGEDNFNLEDMEGFTEFWNNLISDMPL*

>G_Y05_72|OP963314
MSALTTTASSTSSQPPPSLVLLRKGAWTTTEDDLLRKCIEKHGAVRWNRVPQLAGLNRCRKSCRLRWLNYLDPRIKRG
RFEEDEDELIFRLHKLLGNRWSLIAGRLPGRTANDVKNYWNSHLSKKLEAGDKKISRASKSCDTDQTTTSKMVERGKT
EH*H*AATTGLDVEWSISSTRG*CIVGGKFDK***KL*KRKYELSR*RQLQFRGYGRIYRILE*FDI*YATLX

>G_Y07_73|OP963315
MSALTTTASSTSSQPPPSLVLLRKGAWTTTEDDLLRKCIEKHGAVRWNRVPQLAGLNRCRKSCRLRWLNYLDPRIKRG
RFEEDEDELIFRLHKLLGNRWSLIAGRLPGRTANDVKNYWNSHLSKKLEAGDKKISRASKSCDTDQTTTSKMVERGKT
EH*H*AATTGLDVEWSISSTRG*CIVGGKFDK***KL*KRKYELSR*RQLQFRGYGRIYRILE*FDI*YATLX

>G_Y07_74|OP963316
MSALTTTASSTSSQPPPSLVLLRKGAWTTTEDDLLRKCLEKHGAVRWNRVPQLAGLNRCRKSCRLRWLNYLDPRIKRG
RFEEDEDELIFRLHKLLGNRWSLIAGRLPGRTANDVKNYWNSHLSKKLEAGDKKISRASKSCDTDQTTTSKMVERGKT
EH*H*AATTGLDVEWSISSTRG*CIVGGKFDK***KL*KRKYELSR*RQLQFRGYGRIYRILE*FDI*YATLX

>G_Y10_75|OP963317
MSALTTTASSTSSQPPPSLVLLRKGAWTTAEDDLLRKCLEKHGAVRWNRVPQLAGLNRCRKSCRLRWLNYLDPRIKRG
RFEEDEDELIFRLHKLLGNRWSLIAGRLPGRTANDVKNYWNSHLSKKLEAGDKKISRASKSCDTDQTTTSKMVERGKT
EH*H*AATTGLDVEWSISSTRG*CIVGGKFDK***KL*KRKYELSR*RQLQFRGYGRIYRILE*FDI*YATLX

Group 3.2 (Yellow)
>G_P13_10|OP963205
M*ALIIITVSSTSSQPPPSLVLLRWGRWTRTEDDLLRRCIEKHGAVRWNRVPQLAGLNRCRKSCRLRWLNYLDPRIKRE
RFEEDEDDLIFRLHKLLGNRWSLIAGRLPGRTVNDVKNHWNSHLSKKLDAGDQKFRGRRRVATPIRPQPRRWSREEKP
SIDIEQQQQDLTLS*ASAQQKDNALWVERLIRDDENYNNENMNGRPEANFNLVDMEGFTEFWNNLMSDMPL*

>G_P31_32|OP963227
MSALTITVSSTSSQPPPSLVLLRWGRWTRTEDDLLRRCIEKHGAVRWNRVPQLAGLNRCRKSCRLRWLNYLDPRIKRE
RFEEDEDDLIFRLHKLLGNRWSLIAGRLPGRTVNDVKNHWNSHLSKKLDAGDQKFRGRRRVATPIRPQPRRWSREEKP
SIDIEQQQQDLTLS*ASAQQKDNALWVERLIRDDENYNNENMNGRPEANFNLVDMEGFTEFWNNLISDMPL*

>G_Y09_16|OP963258
MSALTIIVSSTSSQPPPSLVLLRRGKWTKTEDDILRRCIEKHGAVRWNRVPQLAGLNRCRKSCRLRWLNYLDPRIKRE
RFEEDEDDLIFRLHKLLGNRWSLIAGRLPGRTANDVKNHWNSHLSKKLDAGDQKFRGRRRVATPIRPQPRTWSREEKP
SINIEQQQQDLTLS*ASAQQKDNALWVERLIRDDENYNNENMNGRPEANFNLVDMEGFTEFWNNLMSDMPL*

>G_Y01_17|OP963259
MSALTITVSSTSSQPPPSLVLLRWGRWTRTEDDLLRRCIEKHGAVRWNRVPQLAGLNRCRKSCRLRWLNYLDPRIKRE
RFEEDEDDLIFRLHKLLGNRWSLIAGRLPGRTVNDVKNHWNSHLSKKLDAGDQKFRGRRRVATPIRPQPRRWSREEKP
SIDIEQQQQDLTLS*ASAQQKDNALWVERLIRDDENYNNENMNGRPEANFNLVDMEGFTEFWNNLMSDMPL*

>G_Y03_18|OP963260
MSALTITVSSTSSQPPPSLVLLRWGRWTRTEDDLLRRCIEKHGAVRWNRVPQLAGLNRCRKSCRLRWLNYLDPRIKRE
RFEEDEDDLIFRLHKLLGNRWSLIAGRLPGRTVNDVKNHWNSHLSKKLDAGDQKFRGRRRVATPIRPQPRRWSREEKP
SIDIEQQQQDLTLS*ASAQQKDNALWVERLIRDDENYNNENMNGRPEANFNLVDMEGFTEFWNNLMSDMPL*

>G_Y15_19|OP963261
MSALTITVSSTSSQPPPSLVLLRWGRWTRTEDDLLRRCIEKHGAVRWNRVPQLAGLNRCRKSCRLRWLNYLDPRIKRE
RFEEDEDDLIFRLHKLLGNRWSLIAGRLPGRTVNDVKNHWNSHLSKKLDAGDQKFRGRRRVATPIRPQPRRWSREEKP
SIDIEQQQQDLTLS*ASAQQKDNALWVERLIRDDENYNNENMNGRPEANFNLVDMEGFTEFWNNLMSDMPL*

>G_Y07_20|OP963262
MSALTITVSSTSSQPPPSLVLLRWGRWTRTEDDLLRRCIEKHGAVRWNRVPQLAGLNRCRKSCRLRWLNYLDPRIKRE
RFEEDEDDLIFRLHKLLGNRWSLIAGRLPGRTVNDVKNHWNSHLSKKLDAGDQKFRGRRRVATPIRPQPRRWSREEKP
SIDIEQQQQDLTLS*ASAQQKDNALWVERLIRDDENYNNENMNGRPEANFNLVDMEGFTEFWNNLMSDMPL*

>G_Y03_27|OP963269
MSALTITVSSTSSQPPPSLVLLRWGRWTRTEDDLLRRCIEKHGAVRWNRVPQLAGLNRCRKSCRLRWLNYLDPRIKRE
RFEEDEDDLIFRLHKLLGNRWSLIAGRLPGRTVNDVKNHWNSHLSKKLDAGDQKFRGRRRVATPIRPQPRRWSREEKP
SIDIEQQQQDLTLS*ASAQQKDNALWVERLIRDDENYNNENMNGRPEANFNLVDMEGFTEFWNNLISDMPL*

>G_Y03_28|OP963270
MSALTIIVSSTSSQPPPSLVLLRRGKWTKTEDDILRRCIEKHGAVRWNRVPQLAGLNRCRKSCRLRWLNYLDPRIKRE
RFEEDEDDLIFRLHKLLGNRWSLIAGRLPGRTVNDVKNHWNSHLSKKLDAGDQKFRGRRRVATPIRPQPRRWSREEKP
SIDIEQQQQDLTLS*ASAQQKDNALWVERLIRDDENYNNENMNGRPEANFNLVDMEGFTEFWNNLMSDMPL*

>G_Y04_32|OP963274
MSALKITVSSTSSQPPPSLVLLRRGMWTKTEDDLLRRCIEKHGAVRWNRVPQLAGLNRCRKSCRLRWLNYLDPRIKRE
RFEEDEDDLIFRLHKLLGNRWSLIAGRLPGRTVNDVKNHWNSHLSKKLDAGDQKFRGRRRVATPIRPQPRRWSREEKP
SIDIEQQQQDLTLS*ASAQQKDNALWVERLIRDDENYNNENMNGRPEANFNLVDMEGFTEFWNNLMSDMPL*

>G_Y14_43|OP963285
MSALTITVSSTSSQPPPSLVLLRWGRWTRTEDDLLRRCIEKHGAVRWNRVPQLAGLNRCRKSCRLRWLNYLDPRIKRE
RFEEDEDDLIFRLHKLLGNRWSLIAGRLPGRTVNDVKNHWNSHLSKKLDAGDQKFRGRRRVATPIRPQPRRWSREEKP
SIDIEQQQQDLTLS*ASAQQKDNALWVERLIRDDENYNNENMNGRPEANFNLVDMEGFTEFWNNLMSDMPL*

>G_Y14_68|OP963310
MSALTITVSSTSSQPPPSLVLLRRGMWTKTEDDLLRRCIEKHGAVRWNRVPQLAGLNRCRKSCRLRWLNYLDPRIKRE
RFEEDEDDLIFRLHKLLGNRWSLIAGRLPGRTANDVKNH*NSHLSKKLDAGDQKFRERRRVATPIRPQPRRWSREEKP
SIDIEQQQQDLTLSRASAQQEDDGLWVERLIRDDENYNNENMNGRPEANFNLEDMEGFTEFWNNLMSDMPL*

References cited in Supplementary information

1. Stracke, R., Werber, M. & Weisshaar, B. The *R2R3-MYB* gene family in *Arabidopsis thaliana*. *Curr. Opin. Plant Biol.* **4**, 447–456 (2001).
2. Zimmermann, I. M., Heim, M. A., Weisshaar, B. & Uhrig, J. F. Comprehensive identification of *Arabidopsis thaliana* MYB transcription factors interacting with R/B-like BHLH proteins. *Plant J.* **40**, 22–34 (2004).
3. Edgar, R. C. MUSCLE: multiple sequence alignment with high accuracy and high throughput. *Nucleic Acids Res.* **32**, 1792–1797 (2004).
4. Tamura, K., Stecher, G., Peterson, D., Filipski, A. & Kumar, S. MEGA6: molecular evolutionary genetics analysis version 6.0. *Mol. Biol. Evol.* **30**, 2725–2729 (2013).
5. Pfaffl, M. W. A new mathematical model for relative quantification in real-time RT-PCR. *Nucleic Acids Res.* **29**, e45 (2001).
